# The lowdown on breakdown: Open questions in plant proteolysis

**DOI:** 10.1093/plcell/koae193

**Published:** 2024-07-09

**Authors:** Nancy A Eckardt, Tamar Avin-Wittenberg, Diane C Bassham, Poyu Chen, Qian Chen, Jun Fang, Pascal Genschik, Abi S Ghifari, Angelica M Guercio, Daniel J Gibbs, Maren Heese, R Paul Jarvis, Simon Michaeli, Monika W Murcha, Sergey Mursalimov, Sandra Noir, Malathy Palayam, Bruno Peixoto, Pedro L Rodriguez, Andreas Schaller, Arp Schnittger, Giovanna Serino, Nitzan Shabek, Annick Stintzi, Frederica L Theodoulou, Suayib Üstün, Klaas J van Wijk, Ning Wei, Qi Xie, Feifei Yu, Hongtao Zhang

**Affiliations:** The Plant Cell, American Society of Plant Biologists, USA; Department of Plant and Environmental Sciences, Alexander Silberman Institute of Life Sciences, Hebrew University of Jerusalem, Jerusalem 9190401, Israel; Department of Genetics, Development and Cell Biology, Iowa State University, Ames, IA 50011, USA; School of Biological Science and Technology, College of Science and Engineering, Kanazawa University, Kakuma-machi, Kanazawa 920-1192, Japan; Ministry of Agriculture and Rural Affairs Key Laboratory for Pest Monitoring and Green Management, College of Plant Protection, China Agricultural University, Beijing 100193, China; Section of Molecular Plant Biology, Department of Biology, University of Oxford, Oxford OX1 3RB, UK; Institut de Biologie Moléculaire des Plantes, CNRS, Université de Strasbourg, 12, rue du Général Zimmer, Strasbourg 67084, France; School of Molecular Sciences, The University of Western Australia, Crawley, Western Australia 6009, Australia; Department of Plant Biology, College of Biological Sciences, University of California-Davis, Davis, CA 95616, USA; School of Biosciences, University of Birmingham, Edgbaston B1 2RU, UK; Department of Developmental Biology, University of Hamburg, Ohnhorststr. 18, Hamburg 22609, Germany; Section of Molecular Plant Biology, Department of Biology, University of Oxford, Oxford OX1 3RB, UK; Department of Postharvest Sciences, Agricultural Research Organization (ARO), Volcani Institute, Rishon LeZion 7505101, Israel; School of Molecular Sciences, The University of Western Australia, Crawley, Western Australia 6009, Australia; Department of Postharvest Sciences, Agricultural Research Organization (ARO), Volcani Institute, Rishon LeZion 7505101, Israel; Institut de Biologie Moléculaire des Plantes, CNRS, Université de Strasbourg, 12, rue du Général Zimmer, Strasbourg 67084, France; Department of Plant Biology, College of Biological Sciences, University of California-Davis, Davis, CA 95616, USA; Section of Molecular Plant Biology, Department of Biology, University of Oxford, Oxford OX1 3RB, UK; Instituto de Biología Molecular y Celular de Plantas (IBMCP), Consejo Superior de Investigaciones Cientificas-Universidad Politecnica de Valencia, Valencia ES-46022, Spain; Department of Plant Physiology and Biochemistry, Institute of Biology, University of Hohenheim, Stuttgart 70599, Germany; Department of Developmental Biology, University of Hamburg, Ohnhorststr. 18, Hamburg 22609, Germany; Department of Biology and Biotechnology, Sapienza Universita’ di Roma, p.le A. Moro 5, Rome 00185, Italy; Department of Plant Biology, College of Biological Sciences, University of California-Davis, Davis, CA 95616, USA; Department of Plant Physiology and Biochemistry, Institute of Biology, University of Hohenheim, Stuttgart 70599, Germany; Plant Sciences and the Bioeconomy, Rothamsted Research, Harpenden AL5 2JQ, UK; Faculty of Biology and Biotechnology, Ruhr-University of Bochum, Bochum 44780, Germany; Section of Plant Biology, School of Integrative Plant Sciences (SIPS), Cornell University, Ithaca, NY 14853, USA; School of Life Sciences, Southwest University, Chongqing 400715, China; State Key Laboratory of Plant Genomics, Institute of Genetics and Developmental Biology, the Innovative Academy of Seed Design, Chinese Academy of Sciences, Beijing 100101, China; University of Chinese Academy of Sciences, Beijing 100049, China; College of Grassland Science and Technology, China Agricultural University, Beijing 100083, China; Plant Sciences and the Bioeconomy, Rothamsted Research, Harpenden AL5 2JQ, UK

## Abstract

Proteolysis, including post-translational proteolytic processing as well as protein degradation and amino acid recycling, is an essential component of the growth and development of living organisms. In this article, experts in plant proteolysis pose and discuss compelling open questions in their areas of research. Topics covered include the role of proteolysis in the cell cycle, DNA damage response, mitochondrial function, the generation of N-terminal signals (degrons) that mark many proteins for degradation (N-terminal acetylation, the Arg/N-degron pathway, and the chloroplast N-degron pathway), developmental and metabolic signaling (photomorphogenesis, abscisic acid and strigolactone signaling, sugar metabolism, and postharvest regulation), plant responses to environmental signals (endoplasmic-reticulum-associated degradation, chloroplast-associated degradation, drought tolerance, and the growth-defense trade-off), and the functional diversification of peptidases. We hope these thought-provoking discussions help to stimulate further research.

## Teaser

Expert opinions on major open questions in plant proteolysis research.

## Introduction

**(Written by Nancy A. Eckardt, Editor)**

Proteolysis is Nature's way of keeping house. While some people can function quite happily in a house full of disorganized piles of a lifetime of accumulated stuff, an organism's ability to thrive and reproduce depends on highly functioning proteolytic systems to keep the “stuff” (i.e. proteins) in check. More than just “housekeeping,” proteolytic systems serve as “house managers”—not only degrading proteins to prevent their overaccumulation and recycle amino acids but also carrying out regulatory processing of proteins to alter or fine-tune critical pathways in growth, development, and responses to environmental signals. Regulation of protein half-life, as well as proteolytic processing as a post-translational modification, is a prevalent mechanism that modulates protein function and ensures proper protein stoichiometries throughout the proteome.

Proteolytic processing occurs through a wide range of mechanisms in eukaryotic cells. In addition to a plethora of individual peptidases located in different cellular compartments, major routes for protein turnover include the ubiquitin-proteasome system (UPS), which operates principally in the cytosol and nucleus, and the delivery of proteins, protein complexes, and organelles to the vacuole for degradation. The two major routes of delivery of cellular components to the vacuole are autophagy and endocytosis. Chloroplasts and mitochondria also maintain independent degradation systems (van Wijk 2015). There are numerous associated pathways for targeting and delivering proteins, protein complexes, and whole organelles bound for degradation to the appropriate destination. In this commentary, researchers working on different aspects of plant proteolysis address major open questions in their field of expertise. We acknowledge that the topics covered represent only a small fraction of the proteolytic events taking place in plant cells, and we apologize to readers whose favorite proteases or proteolytic systems were left out.

### Questions addressed

#### Proteolysis and cell biology

- What is the role of the F-box protein FBL17 in the G1/S-phase transition in Arabidopsis?
- What is the role of autophagy in the plant DNA damage response?
- How do proteolytic networks regulate mitochondrial function?

#### N-terminal signals for degradation pathways

- What is the effect of N-terminal acetylation on protein half-life?
- The Arg/N-degron pathways of protein turnover: Boutique or bulk?
- What are the degrons and molecular players in the chloroplast N-degron pathway?

#### Roles of proteolysis in developmental and metabolic signaling

- How do plants use ubiquitin-mediated proteolysis to regulate photomorphogenesis?
- How does proteolysis of core signaling components occur in different subcellular locations to modulate the abscisic acid (ABA) pathway?
- How does the D14 receptor function as both receptor and enzyme, linking hormone perception to protein degradation?
- Who takes the lead in the intricate dance between autophagy and sugar metabolism?
- What is the role of proteolysis in fruit ripening regulation?

#### Roles of proteolysis in plant responses to biotic/abiotic signals

- How does endoplasmic-reticulum-associated degradation (ERAD) function in model plants and crops?
- How is chloroplast-associated protein degradation (CHLORAD) regulated in response to developmental and environmental cues?
- How does autophagy contribute to drought tolerance?
- How does the fine-tuning of proteasome regulation impact the trade-off between growth and defense?
- Why are there so many peptidases in plants, particularly in the subtilase family?

### The UPS

The UPS tags and delivers proteins to the 26S proteasome, an ATP-dependent, multi-catalytic protease complex that degrades proteins in both the cytoplasm and nucleus (Raffeiner et al. 2023). A specialized pathway of the UPS, ERAD, minimizes the accumulation of damaged or misfolded proteins in the endoplasmic reticulum (ER). Entry to the UPS begins when a protein is modified by the attachment of Ubiquitin (Ub), a 76 amino acid protein that is highly conserved in all eukaryotes. Target proteins are ubiquitylated through the combined activity of E1 Ub-activating enzymes, E2 Ub-conjugating enzymes, and E3 Ub-ligases. The E3 ligase recognizes and binds the target protein. The E1 binds Ub in an ATP-dependent manner and transfers it to an E2. The E2 binds the E3 ligase and transfers Ub to the target protein directly or, for some E3s, Ub is transferred to the E3 and then to the target. Ubiquitylation of a target protein marks it for degradation via the 26S proteasome (Ciechanover et al. 2000). Ub may be attached to a target protein as a monomer or as a linear ubiquitin chain, formed by linkages between one of seven conserved lysine residues. Polyubiquitylation through Ub lysine residue 48 (K48) is one of the main recognition signals for the UPS.

Well over 1,000 E3 ligases have been identified in plants (Al-Saharin et al. 2022; Saxena et al. 2023). Single subunit E3 ligases can be classified into three or four types: HECT (Homologous to E6AP C-Terminus), RING finger (Really Interesting New Gene), U-box (sometimes classified as a subset of the RING-type), and RBR (Ring-Between-Ring). Cullin-RING E3 ligases (CRLs) constitute a single large family of multi-subunit E3 ligases. Plants include all of these types, but the largest family is the CRLs. CRLs are further divided into several different types depending on the cullin (CUL) scaffold (Li et al. 2023b). The largest CRL grouping is the Skp1/Ask1-Cullin1-F-box (SCF) complex, composed of an F-box protein (FBP) that functions in target recognition and three core subunits (CUL1 as the major scaffold unit; RBX1, which binds the E2 Ub-conjugating enzyme; and Skp1/ASK1/2, which recognizes and binds the FBP). The FBP protein defines the SCF complex, and the large number of SCFs is due to the diversity of FBPs. The FBP family represents one of the largest families of regulatory proteins in plants, with many species including hundreds of FBP-encoding genes. For example, the Arabidopsis (*Arabidopsis thaliana*) and rice (*Oryza sativa*) genomes encode ∼700 and 970 FBPs, respectively (Saxena et al. 2023).

### N-degron pathways

In general, substrate proteins carry a degradation signal known as a degron that is sufficient for recognition and degradation by the proteolytic machinery. Degrons are heterogeneous sequences that can be located anywhere in the protein, can act in a *cis* or *trans* mode, and can also be generated by post-translational modifications to specific amino acid residues. N-terminal degrons (N-degrons) are the most studied. They are formed by N-degron pathways, previously referred to as “N-end rule pathways.” Plants have several different N-degron pathways; some of the best studied are the Arg/N- and Ac/N-degron pathways, involving the creation of an N-degron through arginylation or acetylation of the N-terminal residue, respectively (Holdsworth et al. 2020). N-degrons are recognized by other proteins, called N-recognins, specific to each route of degradation. Many N-recognins are Ub E3 ligases, targeting proteins for degradation via the UPS, but links between N-recognins and autophagy have also been reported (Holdsworth et al. 2020).

### Vaculoar degradation

Vacuolar degradation takes place in the large central vacuole called the lytic vacuole, which typically occupies up to 90% of the plant cell volume (Stefan et al. 2013). Proteins and other cargo molecules are transported to the lytic vacuole via multiple routes; the major routes of delivery are autophagy of cytoplasmic cargo (Marshall and Vierstra 2018; Tang and Bassham 2018) and endocytosis of plasma membrane proteins (Valencia et al. 2016). In plants, there are two main types of autophagy: microautophagy and macroautophagy. Macroautophagy is the best understood and involves the formation of a membrane structure called a phagophore around cargo proteins, which develops into a double-membrane autophagosome. The autophagosome outer membrane fuses with the tonoplast and releases the cargo into the vacuole for degradation. In microautophagy, cytoplasmic components are taken up by the vacuole through the invagination of the tonoplast. A third type, known as mega-autophagy (also called autolysis), occurs when vacuolar hydrolases are released directly into the cytoplasm (often the final stage of programmed cell death). In addition to its role in the UPS, Ub is involved in autophagy: whereas K48-linked polyubiquitylation targets proteins for the UPS, K63-linked polyubiquitylation is known, among other functions, to mark cargo for degradation by autophagy (Raffeiner et al. 2023).

Endocytosis regulates the turnover of plasma membrane proteins (such as receptors and transporters), transporting cargo through the endomembrane system in single-membrane vesicles for delivery to the vacuole or recycling back to the plasma membrane (Fan et al. 2015). There is also substantial overlap between these pathways, for example, cross-regulation between the UPS and autophagy pathways (Su et al. 2020b) and between autophagy and endocytosis (Zhuang et al. 2015; Zhang et al. 2019a).

### Peptidases for limited proteolysis

In addition to the proteasome, hundreds of peptidases function in all compartments of the cell including the extracellular matrix, and play roles in almost every aspect of plant development and in the interaction of plants with their biotic and abiotic environment. Among the most abundant peptidases are cysteine, serine, and aspartic proteases, which are named for the amino acid residue that serves as the nucleophile for catalysis, and metalloproteases, which use a polarized water molecule for nucleophilic attack of peptide bonds (van der Hoorn 2008). Some peptidases contribute to protein turnover by nonselective degradation and others perform limited proteolysis of substrate proteins at highly specific sites. Limited proteolysis may result in a loss, gain, or change in activity; it may affect protein assembly and subcellular targeting, and as part of the maturation process, controls the activity of enzymes, regulatory proteins, and signaling peptides (Schaller et al. 2018; Stührwohldt and Schaller 2019). Because peptidases irreversibly modify the structure and function of cognate substrate proteins, their activity is tightly regulated at multiple levels (Fernández-Fernández et al. 2023). Diversification with respect to substrate proteins, cleavage site recognition, and mechanism of regulation may have contributed to the present-day abundance of peptidases in plants, as discussed below for subtilases, one of the largest families of serine peptidases.

## Proteolysis and cell biology: the cell cycle, DNA damage response, and mitochondrial function

### FBL17: a proteolytic engine for the G1/S-phase transition?

**(Written by Pascal Genschik and Sandra Noir)**

Progression through the cell cycle phases depends on cyclin-dependent kinase (CDK) activity (Budirahardja and Gönczy 2009). In plants, this activity is conferred by A- and B-type CDKs, which are activated by multiple cyclins to permit DNA replication and mitosis (Harashima et al. 2013). CDKA; 1 is the main regulator of the G1/S transition, whereas CDKBs are necessary for mitosis (Polyn et al. 2015). At mitotic exit, CDK activity drops and stays low in G1, enabling the licensing of replication origins. This is achieved by several mechanisms working collaboratively, including the selective degradation of mitotic cyclins by the UPS (Mocciaro and Rape 2012). Ubiquitylation of mitotic cyclins is mediated in all eukaryotes by the conserved anaphase-promoting complex or cyclosome (APC/C) Ub E3 ligase (Pesin and Orr-Weaver 2008; Genschik et al. 2014; Willems and De Veylder 2022). CDK activity is also inhibited by the binding of cyclin-dependent kinase inhibitor (CKI) proteins (Besson et al. 2008). In plants, two classes of CKIs carrying distinct functions have been described, called KIP-RELATED PROTEINS (KRPs) and SIAMESE-RELATED proteins [SMRs (Churchman et al. 2006; Acosta et al. 2011)]. It was proposed that KRPs mainly play a role in the G1 checkpoint by inhibiting CDKA; 1–CYCD complexes, whereas SMR members play a prominent role during endoreplication (Van Leene et al. 2010; Kumar et al. 2015).

To re-enter the S-phase and release CDK activity, cells need to decrease the level of CKI proteins. In mammals, the UPS plays a fundamental role in cell cycle control and the DNA damage response (DDR) by destroying CKIs. Two families of mammalian CKIs (INK4 and CIP/KIP) play distinct cellular functions and are degraded by diverse types of E3s in both the nucleus and cytoplasm (Starostina and Kipreos 2012). One of them is the SCF^Skp2^ complex, which plays a prominent role in cell cycle control (Carrano et al. 1999; Sutterlüty et al. 1999; Frescas and Pagano 2008). The F-box protein Skp2 recognizes many substrates involved in cell cycle control and the DDR (reviewed in Frescas and Pagano 2008). Not surprisingly, with such a repertoire of substrates, Skp2 is involved in multiple aspects of different human cancers and is defined as an oncogene (Chan et al. 2010).

With hundreds of publications describing the elaborate multi-task functions of mammalian Skp2, one may wonder whether such a crucial Ub E3 ligase would be conserved in the green lineage. Here we discuss the Arabidopsis F-box protein FBL17, which shows many similarities with mammalian Skp2 (Table), but for which much work is still required to fully grasp its cellular functions. *FBL17* was initially identified as an essential gene needed for male germ cell division in Arabidopsis, with a phenotype similar to the loss of function of *CDKA; 1* (Kim et al. 2008; Gusti et al. 2009). FBL17 appears to function in the degradation of KRPs, supported by the stabilization of KRP6 in *fbl17* single germ cells (Kim et al. 2008), whereas different *krp* mutations at least partially rescued the *fbl17* pollen phenotype (Gusti et al. 2009; Zhao et al. 2012). The function of FBL17 is however not restricted to germ cells.

**Table.** Comparison of characteristics of mammalian Skp2 and Arabidopsis FBL17

Skp2 (mammals)	Characteristic	FBL17 (Arabidopsis)
Yes	Expression during S-phase	Yes
Yes	Transcription targets of E2F/DP	Yes
Viable (mouse)	Loss of function	Gametophyte lethal (with sporophytic escapees)
Reduced cell proliferation	Cell cycle defects in mutants	Reduced cell proliferation
Increase in ploidy	Ploidy in null mutants	Suppressed endoreduplication
p21, p27, p57	CKI as targets	KRPs
Cyclins D1 and E, Cdt1, Orc1, Rbl2/p130, E2F	Other cell cycle targets	?
Yes	Phospho-degron	?
Yes	Requirements of Cks1 cofactor	?
K48, K63, …	Ub chain topology	?
Cdk2, Atk, Wee1	Phosphorylation	WEE1
APC/C^Cdh1^	Degradation	APC/C^CDC20^
ATM kinase activation and DNA DSB repair	Role in DDR	Replication stress and DSBs
Brca2, Nbs1, …	Targets in DDR	?

**Figure 1.**
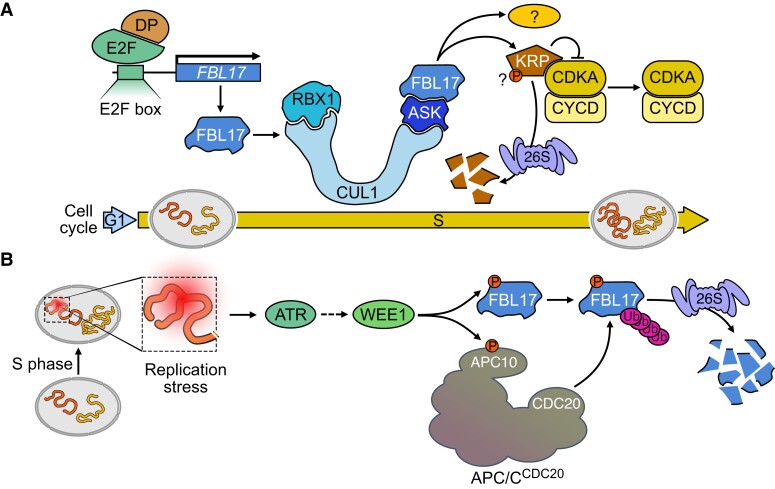
Regulation and possible roles of FBL17 in Arabidopsis. **A)** During the G1/S-phase, E2Fa-DPa directly activates the transcription of *FBL17*. The degradation of KRPs by FBL17-mediated ubiquitylation and degradation would release the activity of CDKA/CYCD. Whether FBL17 recognizes its substrate via a phospho-degron is unknown. **B)** During replication stress, WEE1 phosphorylates FBL17 and the APC10 subunit of the APC/C, which promotes FBL17 ubiquitylation and degradation by the proteasome.

*FBL17* is expressed in the S-phase in synchronized plant cell cultures (Menges et al. 2003; Trolet et al. 2019). In line with this expression pattern, *FBL17* is a direct transcription target of E2Fa-DPa (a transcription factor associated with cell proliferation) and is repressed by the binding of RETINOBLASTOMA-RELATED 1 (RBR1), an Arabidopsis homolog of the Retinoblastoma (Rb) proteins in mammals, to its promoter [Gusti et al. 2009; Zhao et al. 2012 (Fig. 1)]. Viable *fbl17* null mutant plants were identified at very low frequency, and their molecular and cellular characterization revealed major cell cycle defects (Noir et al. 2015). In particular, *FBL17* loss of function drastically reduced cell proliferation and also fully suppressed endoreplication (Noir et al. 2015). Such a phenotype could potentially be explained by a strong accumulation of KRPs that can block S-phase CDK activity (Verkest et al. 2005b), and would be consistent with the phenotypic resemblance of *fbl17* and *cdka; 1* null mutant plants (Nowack et al. 2012; Noir et al. 2015). Accordingly, the KRP2 protein steady-state level was found to increase in *fbl17* mutants (Noir et al. 2015). Another study, using Arabidopsis plants in which *FBL17* function is inhibited by an inducible microRNA, also provided evidence for a degradative role during G2 of the F-box protein in the turnover of free, but not chromosomal bound, KRP4 proteins (D’Ario et al. 2021). Interestingly, meristems with inhibited *FBL17* had abnormally large cells, suggesting that excess free KRP4 disrupts cell size homeostasis. This raises the question of whether *FBL17* loss-of-function phenotypes could be explained solely by an impaired degradation of KRPs. The answer is likely no. Strong KRP2-overexpressing lines not only resemble *flb17* mutant plants in many respects, but also show significant differences. The upregulation of numerous cell cycle and DNA damage genes observed in *fbl17* (Noir et al. 2015; Gentric et al. 2020), also suggests that like mammalian Skp2, FBL17 has a broader range of substrates and functions.

In line with this assumption, it was found that *fbl17* mutants are hypersensitive to double-strand break (DSB)-induced genotoxic stress (Gentric et al. 2020). Note that while in mammals the Rb-related protein p130 is degraded by SKP2 (Tedesco et al. 2002), whether FBL17 also targets plant RBR1 remains unknown. Even in the absence of genotoxic stress, *fbl17* mutants exhibit a higher frequency of DNA lesions and increased cell death in the root meristem. It was further shown that FBL17 colocalizes with RBR1 at DNA damage sites, but its substrates and function at this subcellular location remain unknown (Gentric et al. 2020). It is noticeable that in response to DSBs, mammalian Skp2 is required for the activation and recruitment of the Ataxia-telangiectasia mutated (ATM) kinase to DNA damage foci via non-proteolytic K63-dependent ubiquitylation (Wu et al. 2012). FBL17 was also recently implicated in DNA replication stress, as it was found that the hypersensitivity to hydroxyurea of a null mutant of the ATM and Rad3-related (ATR) kinase can be suppressed by the *fbl17* mutation (Pan et al. 2021b). Importantly, this study revealed that WEE1, a conserved kinase induced by ATR during replication stress (De Schutter et al. 2007), directly phosphorylates FBL17 and promotes its polyubiquitylation and subsequent degradation by the proteasome (Pan et al. 2021b; Fig. 1). Interestingly, human Wee1 is also able to phosphorylate and destabilize Skp2 at least in a human cell line, supporting the conservation of this mechanism (Pan et al. 2021b). It was later shown that FBL17 is ubiquitylated by the APC/C^CDC20^ E3 (Pan et al. 2023) and that WEE1 not only phosphorylates FBL17, but also the APC10 subunit of this Ub E3 ligase, enhancing the interaction between the APC/C substrate adaptor, CDC20, and FBL17. As the chemical inhibition of the APC/C also stabilizes FBL17 in the absence of replication stress (Pan et al. 2023), it seems that this Ub E3 ligase plays a broader role in the post-translational control of FBL17.

Among the ∼700 Arabidopsis F-box proteins known to date, FBL17 is the closest functional homolog to the mammalian Skp2 (Gagne et al. 2002). FBL17 shares with Skp2 many characteristics (Table). Both are direct targets of E2F/DP for a periodic expression during G1/S, both are required for entry in S-phase likely via their ability to degrade CKI proteins, both appear to be phosphorylated by WEE1 and to be substrates of the APC/C, and finally, both are involved in the DDR. Altogether, this makes FBL17 a fascinating protein for further studies of the plant cell cycle and also beyond.

Important questions to tackle in the future include the following. First, and slightly provocative, are KRPs really direct targets of FBL17? Several observations support this conclusion (see above), but to date, none of the *krp* mutations have been reported to suppress the sporophytic phenotype of *FBL17* loss of function. This could be tested with higher-order *krp* mutant combinations. Also, biochemical evidence to demonstrate the direct role of the SCF^FBL17^ in KRP turnover is missing. A fully reconstituted ubiquitylation assay would be valuable. To understand how KRPs are recognized by the Ub E3 ligase is also of great interest. It was shown that the stability of some KRPs, such as KRP2, depends on phosphorylation by CDKs (Verkest et al. 2005a), but whether FBL17 binds a phospho-degron and requires the Cks1 cofactor as Skp2 is currently unknown. Obtaining structural data on the interaction of FBL17 with its substrate would significantly advance the field. The ubiquitylation site on KRPs and the topology of Ub chains should also be addressed.

Second, as Skp2 is reported to target more than a dozen substrates (Frescas and Pagano 2008), we might ask how large is the substrate repertoire of FBL17? Addressing this question is challenging. For instance, if FBL17 recruits its substrates through a phospho-degron, a simple yeast-two-hybrid screening approach to identify new targets may fail. Searching for substrates by either pulldown or proteomics approaches may also be challenging. Since *FBL17* expression is mainly restricted to meristematic tissues, interacting substrates may be of low abundance and difficult to detect. In addition, interactions between F-box proteins and their substrates have been described as versatile, often with low affinity for the substrate (Pierce et al. 2009). Therefore, other techniques such as Ub ligase trapping or proximity labeling should be considered (Iconomou and Saunders 2016).

Finally, it will be necessary to explore the regulation of *FBL17* at both transcriptional and post-translational levels. Given the feature of the G1/S transition as a critical cell cycle checkpoint where multiple signaling pathways are converging, FBL17, by regulating the stability of a number of important players during this transition phase, appears undeniably in a good position to act as a key regulatory node.

### Is autophagy a key process in the plant DNA damage response?

**(Written by Poyu Chen, Maren Heese, and Arp Schnittger)**

The DNA of plant cells, like the DNA of any other organism, is constantly damaged in various ways, including DNA double-strand breaks (DSBs) and DNA cross-links. Upon the detection of damage, a cell launches a specific response called the DNA damage response, which depends on the type and level of the damage experienced as well as on the developmental context and the physiological state of a cell (Chen et al. 2019; Szurman-Zubrzycka et al. 2023). If the DNA is mildly damaged, the DDR usually triggers an arrest of cell proliferation (although DNA replication and cell growth can sometimes continue; Adachi et al. 2011), and a DNA repair program is launched. If the DNA is severely damaged and/or if very little damage is tolerated due to developmental constraints, such as in stem cells (Fulcher and Sablowski 2009), terminal differentiation or death of the damaged cell will be induced (Chen et al. 2019). These cellular responses rely on a specific transcriptional response in which the NAC transcription factor SUPPRESSOR OF GAMMA RADIATION1 (SOG1) plays a central role by inducing the expression of, for instance, genes repressing cell division, such as the CYCLIN-DEPENDENT KINASE inhibitors SIAMESE-RELATED PROTEINS 5 and 7 (SMR5 and SMR7), and genes involved in the actual mending of DNA, such as the recombinase RADIATION SENSITIVE 51 (RAD51) and CYCLINB1; 1 (CYCB1; 1), which both are involved in homologous recombination (HR) repair (Bleuyard et al. 2005; Yoshiyama et al. 2009; Yi et al. 2014; Weimer et al. 2016; Ogita et al. 2018).

**Figure 2.**
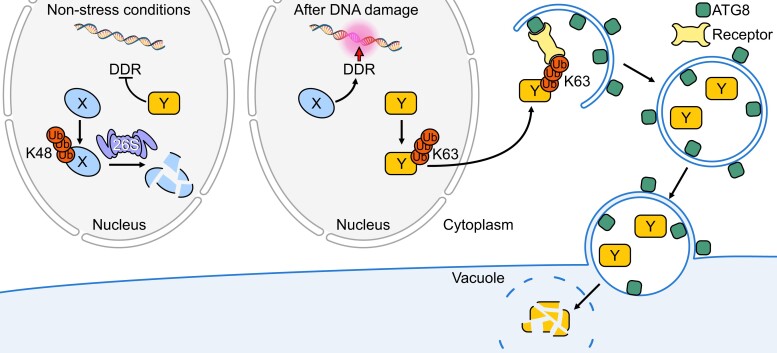
Proteasome- and vacuole-dependent protein degradation in the plant DDR. After DNA damage, a plant cell launches a DNA damage response (DDR), which includes the transcriptional upregulation of certain genes and the targeted degradation of proteins. The two major protein degradation systems, the UPS and the vacuole-dependent system (autophagy) both appear to be involved in the DDR. The diagram shows a prototypical proteasome target (green X). Under non-stress conditions (gray arrows), X is marked by K48 polyubiquitin chains and subsequently degraded by the proteasome. After DNA damage, X becomes stabilized and participates in the DDR. Examples of X are the transcriptional repressor of cell proliferation MYB3R3 and the RMI1 removing factor KNO1. Another and previously not DDR-associated degradation pathway is shown for the orange-marked protein Y, a protein that interferes with efficient DDR. Under non-damaging conditions, Y is present. Under damaging conditions, Y is polyubiquitylated via K63 chains marking it for autophagy-dependent degradation via the cytoplasm. The only example for Y so far is the RTR-complex scaffolding component RMI1, which becomes polyubiquitylated via K63, targeting it for removal to the cytoplasm and degradation via autophagy. The observation that mutants in the macroautophagy pathway (such as ATG mutants) are sensitive to various DNA-damage-inducing drugs indicates that macroautophagy also degrades other proteins after DNA damage and likely plays a major role in the DDR of plants.

However, targeted degradation of proteins also plays a pivotal, yet so far not well-studied role in the DDR of plants. In general, the removal of specific proteins can be executed by two different systems: (1) the proteasome, present in the nucleus and the cytoplasm, and (2) selective autophagy, i.e. degradation via lytic compartments such as the vacuole in plants and the lysosome in animal cells, executed in the cytoplasm (Fig. 2). Previously, proteasome-mediated protein degradation has been implicated in the DDR of Arabidopsis; for example, the transcriptional repressor MYB3R3, which is involved in cell cycle arrest after DNA damage, is blocked from proteasomal degradation under DNA damaging conditions (Chen et al. 2017b). Conversely, the mitotic regulator CDKB2; 1 becomes degraded in a proteasome-dependent manner upon DSB induction (Adachi et al. 2011). In contrast, it was not known until recently whether autophagy, which in plants is subdivided into the three forms of microautophagy, macroautophagy, and mega-autophagy (Marshall and Vierstra 2018), is involved in a plant's response to DNA damage.

Autophagy has emerged as an important regulatory mechanism of cellular homeostasis in many, if not all, eukaryotes and for animals, there is evidence that different types of autophagy are also involved in the DDR. However, the picture is still fragmented, and the identity of the specific autophagy targets remains for the most part enigmatic (Juretschke and Beli 2021). Recently, macroautophagy was identified to play a central role in plant DDR, i.e. during DNA cross-links repair in Arabidopsis, where it was shown to be required for the selective removal of a repressor of HR (Chen et al. 2023b). HR in plants is usually repressed during somatic growth and development by the action of the RTR complex, homologous to the BLOOM syndrome complex (BTR complex) in animals. The central components of this complex are a RECQ-type helicase and the topoisomerase TOP3alpha attached to a scaffolding protein named RMI1 (Hartung et al. 2008). To allow for elevated HR after DNA cross-link induction, RMI1 was now shown to be removed in a macroautophagy-dependent manner (Chen et al. 2023b).

K63-linked polyubiquitylation (in contrast to K48 polyubiquitin chains that mark proteins for proteasome-dependent degradation) is known, among other functions, to mark cargo for degradation by autophagy (Tan et al. 2008; Nathan et al. 2013). KNOTEN1 (KNO1) of Arabidopsis, a nuclear protein previously implicated in the DDR (Bouyer et al. 2018), was now found to be required for the attachment of K63-linked polyubiquitin to RMI1, which subsequently leads to RMI1 degradation in the cytoplasm in a lytic compartment-derived manner [Chen et al. 2023b (Fig. 2)]. Interestingly, KNO1 itself is also a target of selective protein turnover and was found to be degraded under non-DNA damaging conditions by the proteasome (Chen et al. 2023b). Thus, proteasomal and vacuolar degradation systems appear to be tightly interconnected and collaborate during DDR.

Notably, the impact of macroautophagy on DDR likely goes far beyond the regulation of RMI1. Macroautophagy relies on a group of autophagy-related (ATG) proteins that regulate the formation of autophagosomes and promote their delivery to the vacuole (Su et al. 2020a). Analysis of Arabidopsis mutants of the central autophagy components *ATG2*, *ATG5*, and *ATG7* revealed that all three mutants are not only sensitive to the DNA cross-link inducing agent cisplatin but also to drugs that cause other types of DNA damage, i.e. hydroxyurea (HU), which interferes with DNA replication and produces single-stranded DNA, as well as zeocin, which induces DNA double-strand breaks (Chen et al. 2023b). Since mutants in *KNO1* are particularly sensitive to DNA cross-linkers, but not to HU or DNA double-strand inducing drugs (Bouyer et al. 2018), it seems likely that *KNO1* independent routes exist, that target proteins to macroautophagy after DNA damage and that several proteins are removed by macroautophagy during the DDR. Autophagy has been found to function not only in a pro-survival (Torii et al. 2016) but also in a cell death-promoting manner in humans (Liu et al. 2018b) and plants (Kabbage et al. 2017; Üstün et al. 2017). Thus, it seems possible that autophagy in plants is also involved in a wider context of DDR, e.g. possibly by controlling the cell death response.

### How do proteolytic networks regulate mitochondrial function?

**(Written by Abi S. Ghifari and Monika W. Murcha)**

**Figure 3.**
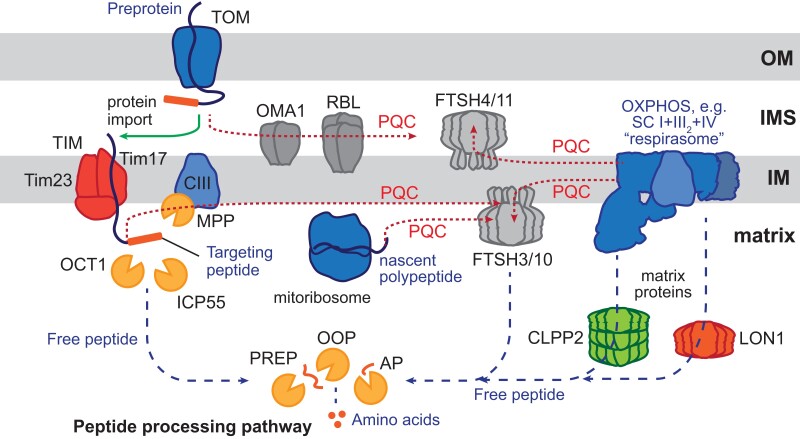
Proteolytic networks across different plant mitochondrial compartments. Protein quality control (PQC in red dotted lines) that includes the disassembly, unfolding, and degradation of proteins and complexes is carried out by various proteases as indicated. PQC is carried out at various pathways including protein import, organello translation, and assembly. Preproteins are imported from the cytosol through the translocase of the outer membrane (TOM) complex. Preproteins targeted to the outer membrane (OM) and the IMS undergo PQC by various IMS-facing proteases, such as overlapping with m-AAA protease OMA1 and rhomboid-like (RBL) protease. Preproteins targeted to the inner membrane (IM) and the matrix are translocated by the translocase of the inner membrane (TIM) complex. Preproteins then undergo the maturation process to remove the N-terminal targeting signal and destabilizing residues by mitochondrial processing peptidase (MPP) embedded within Complex III (CIII) in plants, octapeptidyl peptidase OCT1, and intermediate cleavage peptidase ICP55. Proteins assembled within a complex, such as the oxidative phosphorylation (OXPHOS) supercomplex SC I + III_2_ + IV (or the respirasome) may undergo PQC by the IM-embedded proteases like the matrix-facing FTSH3/FTSH10 and the IMS-facing FTSH4/FTSH11. Nascent polypeptides translated by the mitoribosome may be regulated by FTSH3/10, while PQC of matrix-facing and matrix-located proteins is regulated by the matrix-located CLPP2 and LON1 proteases. Free peptides generated from both targeting peptide cleavage and protein degradation (blue dashed lines) can be further degraded into single amino acids in the peptide processing pathway. This multi-step pathway uses multiple peptidases, including presequence peptidases (PREP), organellar oligopeptidase (OOP), and various aminopeptidases (AP), including alanyl aminopeptidase (AAP), leucyl aminopeptidase (LAP), aspartyl aminopeptidase (DAP), and prolyl aminopeptidase (PAP).

Mitochondria are central organelles, responsible for vital biochemical pathways, including aerobic respiration and biosynthesis of amino acids, lipids, and redox cofactors, among many other functions (Spinelli and Haigis 2018). These pathways rely on the homeostasis of thousands of mitochondrial proteins that are maintained through continuous transcription and translation of nuclear and mitochondrial genomes, protein import, assembly, and finally, degradation of damaged and aggregated proteins (Vazquez-Calvo et al. 2020). Proteolysis plays a role at all stages of mitochondrial biogenesis, from the onset, with regards to protein synthesis and assembly, to protein turnover and degradation (van Wijk 2015; Ghifari and Murcha 2022). Evidence suggests that there is a network of proteases with overlapping activities in organelles (van Wijk 2015; Majsec et al. 2017). These proteolytic networks regulate protein function and abundance to maintain protein homeostasis across various mitochondrial compartments and functions (Fig. 3).

What role do proteases play in protein import and maturation? Most proteins destined for mitochondria are synthesized with cleavable N-terminal targeting peptides that initiate protein translocation through the Translocase of the Outer Membrane (TOM) complex (Fig. 3; Pfanner et al. 2019). Upon import, proteins are matured by various peptidases, such as the mitochondrial processing peptidase (MPP), octapeptidyl peptidase 1 (OCT1), and intermediate cleavage peptidase 55 (ICP55) to cleave the N-terminal targeting peptides and any subsequent unstable residues (Carrie et al. 2015; Huang et al. 2015; Gomes et al. 2017). A distinguishing feature of plant mitochondria is that the a and b subunits of MPP are also integral components of the cytochrome *bc_1_* complex (Complex III) of the respiratory oxidative phosphorylation (OXPHOS) system (Emmermann et al. 1993; Glaser et al. 1994). The enzymatic activity of MPP/*bc1* is independent of electron transfer (Eriksson et al. 1996) and recent structural studies of Complex III_2_ have shown that the a and b subunits of MPP form a large cavity allowing for presequence binding (Maldonado et al. 2021). The distinctive dual function of MPP in plants may be a mechanism of regulating protein import with the requirement for substrates, particularly the subunits of the electron transport chain. Proteases have also been implicated in maintaining the abundance of the protein import machinery, which in turn regulates protein uptake rates (Lister et al. 2007; Wang et al. 2012). For example, immunoprecipitation experiments have identified Tim17-2, the inner membrane transporter channel protein as a substrate of Filamentous Temperature Sensitive-H 4 (FTSH4) in Arabidopsis (Opalinska et al. 2018).

The initial cleavage via MPP generates peptides with the potential to disrupt membrane integrity and inhibit protein import (Zardeneta and Horowitz 1992; Hugosson et al. 1994). Mitochondrial targeting peptides are further degraded in a multi-step peptide processing pathway by numerous matrix-located proteases with overlapping specificity such as the presequence peptidase (PREP) and organellar oligopeptidase [OOP (Bhushan et al. 2005; Ståhl et al. 2005; Kmiec et al. 2013)]. Single amino acids are recovered from short peptides by various aminopeptidases [AP (Waditee-Sirisattha et al. 2011; Kmiec et al. 2018b; Ghifari et al. 2020)]. Plant mitochondria contain at least 15 individual peptidases involved in the process of removing the targeting signal and processing it into single amino acids (Ghifari et al. 2019). Interestingly, most of these peptidases are dually targeted to both mitochondria and chloroplasts, demonstrating a common bacterial-derived peptidolytic network (Kmiec et al. 2018b), alongside distinct protein import mechanisms. The activities of the dual-targeted intermediate peptidases PREP and OOP are most strikingly observed in chloroplasts whereby functional losses of these peptidases led to an accumulation of peptides of chloroplast origin (Kmiec et al. 2018a; Rowland et al. 2022). However, the effect may be more subtle in mitochondria and has yet to be observed.

What proteases are involved in maintaining mitochondrial protein quality control (PQC)? General mitochondrial proteolytic networks primarily composed of ATP-independent proteases such as degradation of periplasmic protein (DEG) and rhomboid-like (RBL; García-Lorenzo et al. 2006) and ATP-dependent such as members of the ATPase-associated with various cellular activities (AAA+) family, which includes FTSH proteases, caseinolytic proteases (CLP), and LON (long filamentous phenotype) proteases [Puchades et al. 2020; Heidorn-Czarna et al. 2022 (Fig. 3)]. Mitochondrial inner membrane proteins are maintained by the matrix-facing (m-AAA) FTSH3 and FTSH10 and the intermembrane space (IMS)-facing (i-AAA) FTSH4 and FTSH11 (Kolodziejczak et al. 2007, 2018, Janska et al. 2010; Heidorn-Czarna et al. 2018; Maziak et al. 2021). Matrix-located AAA+ proteases, such as CLPP2 and LON1, are active toward both soluble matrix and matrix-facing membrane-bound proteins (Li et al. 2017; Petereit et al. 2020). OMA1 (overlapping activity with m-AAA protease-1) primarily maintains the outer membrane (OM) and the IMS proteins (Migdal et al. 2017; Gilkerson et al. 2021).

How are OXPHOS complexes turned over? OXPHOS complexes are large, multi-subunit, dynamic complexes of the inner membrane capable of forming larger supercomplex structures (Schlame 2021). Composed of both nuclear- and mitochondrial-encoded subunits, OXPHOS complexes require intricate coordination, assembly, and regulation (Vercellino and Sazanov 2022; Ghifari et al. 2023b). Furthermore, individual subunits are differentially susceptible to oxidative damage exhibiting distinctive protein turnover rates (Li et al. 2017; Szczepanowska et al. 2020). This suggests that submodules and domains are disassembled and degraded in a modular fashion. A recent study demonstrated that the ATPase domain of FTSH3 promotes the disassembly of the Complex I matrix arm domain (Ivanova et al. 2021) by directly interacting with a specific Complex I subunit (Ghifari et al. 2023a). Structures of FTSH3 homologs revealed that this domain can recognize and bind elongated peptides for degradation (Puchades et al. 2017, 2019). When damaged or misfolded, proteins expose their N-terminal peptide, which serves as a degradation signal that can be recognized by the ATPase domain of AAA+ proteases (Rampello and Glynn 2017).

This function of FTSH3 has only so far been associated with Complex I, yet all OXPHOS complexes are continually undergoing disassembly and turnover. The challenge lies ahead in discovering the mechanisms of substrate recognition and disassembly. Furthermore, the interconnectivity and how the protease functions are coordinated remains unknown. One of the biggest challenges in using single loss-of-function mutants is that often these knockout plants display mild phenotypic change and subtle biochemical changes, due to overlapping functions or gene duplication (Kmiec et al. 2014; Petereit et al. 2020). Whilst a functional interconnection between proteolytic and peptidolytic degradation has been well demonstrated in chloroplasts (Rowland et al. 2022), the interconnectivity of mitochondrial proteolytic networks needs further experimental confirmation. Identification of substrates, interactors, and proteins in proximity using mass spectrometry-based methods can also provide a more comprehensive view. Trapping approaches, whereby the catalytic function of protease is nullified to trap the protein substrate, have revealed potential substrates and specific activities of various proteases (Opalińska et al. 2017; Heidorn-Czarna et al. 2018; Rei Liao and van Wijk 2019). Proximity-based techniques, such as biotinylation and chemical crosslinking in yeast have also revealed that the prohibitin/m-AAA protease complex is in proximity to both translation machinery and protein import complexes, demonstrating its importance in determining the fate of newly synthesized and newly imported proteins (Singh et al. 2020; Kohler et al. 2023). A combination of these techniques may provide a more comprehensive view and a better understanding of the modulation and interconnectivity of proteases in plant mitochondria.

## N-terminal signals for degradation pathways

### To destroy or not to destroy? What is the effect of N-terminal acetylation on protein half-life?

**(Written by Daniel J. Gibbs)**

Protein N-terminal (Nt-)acetylation (NTA) involves the transfer of acetyl moieties from acetyl-coenzyme A to the α-amino group of Nt-amino acid residues by enzymes called Nt-acetyltransferases [NATs (Ree et al. 2018; Aksnes et al. 2019; Giglione and Meinnel 2021)]. This modification occurs on 60% to 80% of all proteins in eukaryotes and is assumed to be irreversible, since no Nt-deacetylases are known (Giglione and Meinnel 2021). Until recently, NTA was thought to be exclusively and constitutively imprinted during mRNA translation by ribosome-tethered NATs and unlikely to play a significant regulatory role in protein function and signaling. However, recent studies in plants have shown that NTA can occur post-translationally within plastids and that the activities of certain NATs are linked to abiotic, biotic, and cellular stress responses (Linster et al. 2015; Bienvenut et al. 2020; Huber et al. 2020; Huber et al. 2021). Dual NTA and internal Lysine-acetylation activities have also been reported for some acetyltransferases, broadening our knowledge of acetylation complexity and crosstalk (Bienvenut et al. 2020). By neutralizing the positive charge of the α-amino group, NTA bestows new biochemical properties that can directly affect protein folding, avidity for protein-interaction partners, subcellular targeting, and protein stability. Here I discuss current knowledge on the complex relationship between NTA and proteolysis via the UPS, with a particular focus on seemingly contradictory findings as well as key open questions in the field.

**Figure 4.**
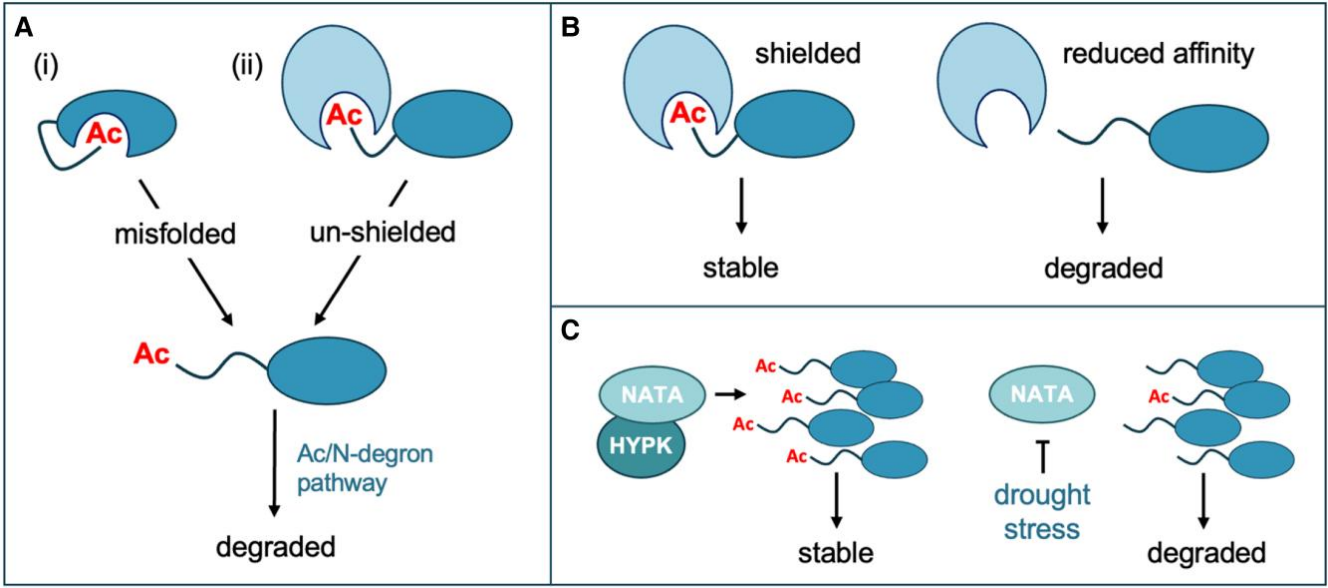
The relationship between NTA and protein stability. **A)** The conditionality of Ac/N-degrons and their link to protein quality control. Acetylated (Ac) N-termini are often shielded through internal protein folding (i) or protein–protein interactions (ii) but can be exposed through protein misfolding or if there is an excess of a particular protein complex subunit. This leads to exposure of the acetylated N-terminus, which can act as a specific degron for proteasomal degradation via the Ac/N-degron pathway (Shemorry et al. 2013). **B)** Hypothetical indirect effects of NTA on protein stability. NTA can increase protein-interaction affinities, to create more stable complexes. A lack of NTA can lead to reduced thermostability, complex breakdown, and the consequent degradation of non-bound and potentially misfolded subunits via then UPS (e.g. as has been shown for cytosolic ribosomes in yeast; Guzman et al. 2023). **C)** NATA-mediated NTA (potentiated by HYPK in plants and mammals) was shown to promote broad proteome stabilization in diverse eukaryotic taxa. In plants, drought-induced downregulation of NATA activity leads to reduced NTA of NATA substrates and an increase in their degradation via exposed “non-Ac/N-degrons” (Linster et al. 2015, 2022). This suggests that NATs may integrate stress signals to control proteome turnover.

In the early 2010s, several studies in yeast and mammals demonstrated that NTA can directly target proteins for degradation, via an acetylation-dependent branch of the N-degron pathway (Ac/N-degron pathway) (Hwang et al. 2010; Shemorry et al. 2013; Gibbs et al. 2014; Park et al. 2015). Here, the N-termini of Nt-acetylated protein substrates are recognized and ubiquitylated by E3 ligases called Ac/N-recognins, which include DOA10/TEB4 and NOT4. A diverse but constrained set of Ac/N-degron pathway substrates was identified, and crucially it was shown that Ac/N-degrons are conditional, since they are usually shielded by protein folding or through intermolecular sequestration (Shemorry et al. 2013). Thus, it was proposed that Ac/N-degrons might contribute to protein quality control by allowing the recognition and rapid elimination of misfolded proteins or excess subunits of multi-protein complexes (Fig. 4A; Nguyen et al. 2018). In contrast to this view, loss of NTA on yeast ribosomal proteins was shown to reduce overall ribosome thermostability, leading to an increase in subunit degradation via the UPS (Guzman et al. 2023). This raises a key question as to whether NTA indirectly influences protein turnover through its effects on protein–protein interaction affinities (Fig. 4B).

There are several reports of plant proteins that are directly destabilized due to NTA, implying potential conservation of the Ac/N-degron pathway in this lineage. This includes a particular Nt-variant of the immune receptor SNC1 that is acetylated by NATA (Xu et al. 2015b), as well as OsHYPK in rice, itself a substrate, interaction partner, and potentiator of NATA activity (Gong et al. 2022). In neither case was the cognate E3 ligase identified. A recent study investigated potential roles for putative Arabidopsis DOA10 homologs as Ac/N-recognins but found no clear connection between DOA10 function and the turnover of Nt-acetylated proteins (Etherington et al. 2023). As such, E3 ligases that recognize Nt-acetylated N-termini in plants await discovery. Interestingly however, cross-species analyses did show kingdom-specific differences in the effect of NATs on the stability of the same protein target through indirectly promoting protein turnover, perhaps through influencing other E3 ligases or the proteasome.

Paradoxically, NTA has also been directly linked to increased stability of specific plant proteins, including SIB1, a positive regulator of salicylic acid–induced cell death, and an alternative N-terminal variant of SNC1 targeted by NATB (Xu et al. 2015b; Li et al. 2020b). This latter finding is particularly intriguing as it highlights how NTA of two different Nt-variants of the same protein can either increase or decrease protein half-life (Gibbs 2015; Xu et al. 2015b). Larger scale studies in yeast, mammalian cells, and plants have also revealed that NATA-mediated NTA is broadly associated with proteome stabilization (Mueller et al. 2021; Gibbs et al. 2022; Guzman et al. 2023). Loss of NATA or HYPK function in Arabidopsis and rice led to increased turnover rates of NATA substrate proteins, which was accompanied by a compensatory increase in translation rates of the same proteins, mediated via the target of rapamycin (TOR) kinase (Linster et al. 2022; Miklankova et al. 2022). This points to the presence of “non-Ac/N-degrons” that are exposed only when NATA activity is downregulated, for example during drought (Fig. 4C; Linster et al. 2015). As such it was posited that regulation of protein NTA might be crucial for rapid proteome turnover to replenish protein pools in response to certain stresses that impact NAT function.

How this might occur is yet to be determined, but the concept of “N-degron complementarity” was previously proposed, whereby obstruction of one pathway can redirect a substrate to a different pathway (e.g. a lack of NTA might instead allow targeting via the Arg/N-degron pathway) (Park et al. 2015; Nguyen et al. 2018). Indeed, this was recently demonstrated for different NATB and NATC substrates in mammals, where NTA was shown to prevent degradation by the Arg/N-degron pathway E3 ligase UBR4 (Guedes et al. 2023; Varland et al. 2023). A study in yeast also showed that NTA can stabilize proteins independent of their ubiquitylation, suggesting that additional proteolytic pathways must be considered (van de Kooij et al. 2023). Moreover, different mechanisms are probably at play in different eukaryotic kingdoms. For example, the IAP E3 ligases shown to bind non-acetylated NATA protein substrates in mammalian cells are not found in plants (Mueller et al. 2021).

Despite its prevalence, NTA remains a somewhat enigmatic modification without a single defined effect on protein stability, although there is increasing evidence that the “default” effect is to promote stability, while still being able to trigger degradation of a more restricted set of specific proteins. Thus, NTA seems to influence protein half-lives in a substrate and context-specific manner. Several key questions linked to the study of NTA and its effects on proteolysis remain: (1) Does NTA influence protein half-life co-translationally, post-translationally, or both? (2) Are NTA-mediated effects on protein stability direct or indirect, and can different NATs have contrasting effects on substrate turnover? (3) Does NTA catalyzed by other NATs (in addition to NATA) also trigger large-scale protein stabilization in plants, and is this linked to shielding against the Arg/N-degron pathway? (4) Does the partial acetylation observed for some substrates act as a switch to flexibly control protein half-lives? (5) Does NTA have a broader role to play in nascent proteome remodeling in response to signals that affect NAT function? (6) What is the significance of post-translational NTA in plastids, and does it contribute to protein degradation in these or other organelles? By focusing on these questions, the stage is set to provide new insight and further clarity into roles for this widespread protein modification in cellular proteostasis.

### The Arg/N-degron pathways of protein turnover: boutique or bulk?

**(Written by Frederica L. Theodoulou and Hongtao Zhang)**

The Arg/N-degron pathway was first defined in the context of arginylation, but now effectively refers to all non-acetylated N-degrons (Varshavsky 2019). Intriguingly, evidence to date indicates that in plants, Arg/N-degron pathways predominantly target short-lived regulatory proteins with unacetylated N-termini, whereas a much wider range of cellular/proteostatic functions has been reported for animals and yeast. Moreover, whilst a seminal study quantifying the half-lives of artificial reporter proteins in yeast established the concept of “stabilizing” and “destabilizing” Nt residues, it is now evident that all 20 proteogenic amino acids can potentially act as Nt degradation signals (N-degrons) in non-plant systems (Bachmair et al. 1986; Varshavsky 2019). This raises the question of how many undetected substrates and processes are regulated by the Arg/N-degron pathways in plants and how these contribute to plant physiology.

#### Do we know all the players?

**Figure 5.**
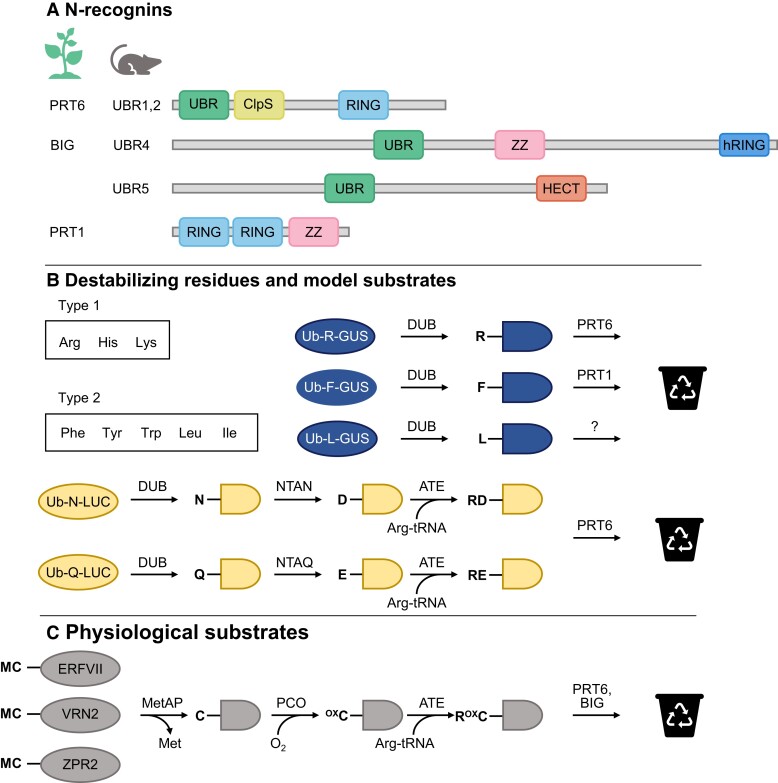
Enzymes and substrates of plant Arg/N-degron pathways. **A)** Schematic of N-recognins in plants and mammals showing substrate recognition and E3 ligase domains (drawn approximately to scale; hRING, hemi-RING). **B)** Destabilizing residues and specificity of Arabidopsis N-recognins, deduced from model substrates (Garzón et al. 2007; Graciet et al. 2010). In these examples, Ub fusion protein is cleaved in planta by deubiquitylating enzymes (DUBs) to produce glucuronidase (GUS) or luciferase (LUC) with Nt destabilizing residues (shown in single letter amino acid code). Proteins with primary destabilizing residues (R, F, L) are targeted for proteasomal degradation by known and unknown N-recognins. Tertiary destabilizing residues N and Q are converted to D and E by Nt(Asn) amidase (NTAN) and Nt(Gln) amidase (NTAQ), respectively. These secondary destabilizing residues are then Nt-arginylated by arginyl-tRNA-transferases (ATEs), enabling PRT6-mediated degradation. **C)** Physiological substrates of the Arg/N-degron pathway. Met-Cys-initiating proteins VRN2, ZPR2, and ERFVIIs undergo co-translational Met excision by methionine amino peptidases (MetAPs) to reveal Nt-Cys, a tertiary destabilizing residue. Following Nt oxidation by plant cysteine oxidases (PCOs) and arginylation by ATEs, they become substrates for PRT6. BIG also mediates the degradation of ERFVIIs and VRN2.

Arg/N-degrons are revealed by proteolytic cleavage, or created by a subsequent enzymatic modification to produce destabilizing N-termini that are recognized by Ub E3 ligases (known as N-recognins) and targeted for proteasomal degradation (Fig. 5). Plastids and mitochondria do not have an internal UPS but are proposed to house discrete N-degron pathways employing the Clp AAA+ protease system (Bouchnak and van Wijk 2019; see next section by van Wijk). The architecture of the nuclear-cytosolic Arg/N-degron pathway and destabilizing residue identities are broadly conserved between yeast, mammals, and plants but plants have a unique complement of N-recognins (Garzón et al. 2007; Graciet et al. 2010). Mammalian N-recognins have overlapping specificity for different classes of destabilizing residues (Type 1, basic; Type 2, aromatic, bulky) and act semi-redundantly (Tasaki et al. 2005). They share a Ub amino-end recognizing (UBR) box but also contain additional motifs involved in substrate recognition and different E3 ligase domains: RING in UBR1 and 2, HECT in UBR5, and a non-canonical hemi-RING E3 domain in UBR4 (Tasaki et al. 2005; Barnsby-Greer et al. 2024).

In contrast, the Arabidopsis homolog of UBR1/2, PROTEOLYSIS6 (PRT6) targets Type 1 N-termini but lacks the ClpS-like domain of UBR1/2 that acts as a recognition domain for Type 2 residues (Garzón et al. 2007). This function has been replaced in the green plant lineage by PROTEOLYSIS1 (PRT1), a unique protein with two RING fingers and a ZZ domain (Potuschak et al. 1998; Till et al. 2019). Although this suggests that separating turnover of Type 1 and 2 substrates could have adaptive value in plants, BIG, an Arabidopsis homolog of UBR4, has recently been implicated in the degradation of substrates with both basic and aromatic N-termini (Zhang et al. 2024a). Plant genomes lack a UBR5 homolog, and it is clear from protein stability reporter studies that further N-recognins remain to be identified, including the elusive Nt-Leu/Ile recognition component(s) (Garzón et al. 2007; Graciet et al. 2010). Whilst genetic evidence strongly supports a role for PRT6 and BIG as N-recognins, biochemical characterization of these very large proteins is challenging and E3 activity has only been formally demonstrated for PRT1 (Stary et al. 2003; Mot et al. 2018).

An important related question is how N-recognins partner with different E2 enzymes and whether they assemble different Ub linkages, potentially with different cellular outcomes (Brillada and Trujillo 2022; Orosa-Puente and Spoel 2022). Here, reconstitution of the pathway in yeast has provided valuable first insights (Kozlic et al. 2022) and the molecular basis of substrate recognition and ubiquitylation will also be informed by advances in structure determination and predictions (Pan et al. 2021a; Sherpa et al. 2022; Jeong et al. 2023; Barnsby-Greer et al. 2024). Structural studies may also shed light on Arg/N-degron pathway-proteasome complexes recently identified by biochemical approaches (Oh et al. 2020b; Zhang et al. 2024a).

#### Do we know all the substrates?

Thus far, only a handful of Arg/N-degron pathway substrates have been identified in plants (Holdsworth et al. 2020). This is in stark contrast to animals and yeast, where the Arg/N-degron pathways participate in cytosolic protein quality control, including degradation of misfolded proteins, mistranslocated proteins, and retrotranslocated organellar proteins, as well as the control of protein subunit stoichiometry (Varshavsky 2019). At present, there is little evidence for this in plants.

The majority of substrates confirmed in planta are Met-Cys-initiating proteins, comprising Group VII ETHLENE RESPONSE FACTOR transcription factors (ERFVIIs), the polycomb repressive complex 2 subunit, VERNALIZATION2 (VRN2), and the LITTLE ZIPPER 2 (ZPR2) transcription factor (Gibbs et al. 2011, 2018; Licausi et al. 2011; Weits et al. 2019). Following co-translational cleavage of Met1 by aminopeptidases, Cys2 may be converted to Cys-sulfinic acid by PLANT CYSTEINE OXIDASE (PCO) enzymes (Weits et al. 2014; White et al. 2017), rendering the protein susceptible to arginylation and PRT6-mediated proteasomal degradation (Fig. 5). Recent evidence suggests the potential presence of further enzymes contributing to complete Nt-Cys oxidation (Zubrzycki et al. 2023). Thus, oxygen-dependent turnover of regulatory proteins by the Arg/N-degron pathways plays a central role in environmental and developmental hypoxia sensing (Holdsworth et al. 2020). Characterization of *prt6* and *ate* mutant plants has revealed further functions of the Arg/N-degron pathways in a/biotic stress responses and development; interestingly, the majority of these are attributable to the regulation of ERVIIs (Holdsworth et al. 2020).

Nevertheless, other substrates must exist: PRT6-dependent, ERFVII-independent control of hypoxia-responsive genes has been reported (Zubrzycki et al. 2023), and conservation of arginyl-tRNA-transferase and Nt amidase specificity in plants implies the existence of PRT6 substrates that are not Met-Cys proteins [Fig. 5 (Graciet et al. 2010; Vicente et al. 2019)]. However, these cannot easily be predicted. An N-degron comprises not only the Nt residue but also appropriately positioned Lys residues for Ub conjugation, which must both be sufficiently accessible to N-recognins (Varshavsky 2019). Accordingly, not all Met-Cys proteins are N-degron pathway substrates (Gibbs et al. 2011; Bäumler et al. 2019; Kozlic et al. 2022), nor are all proteins with other Nt destabilizing residues revealed through endopeptidase cleavage (e.g. RIN4; Goslin et al. 2019; Kozlic et al. 2022).

Mutants impaired in Arg/N-degron function grow like wild-type plants under non-stressed conditions (except for *big* alleles which are pleiotropic), implying that phenotypes- and substrates- may be cryptic. Proteases act as gatekeepers of the Arg/N-degron pathway and offer a largely unexplored route to substrate identification through protein N-terminome (“degradome”) analysis (Perrar et al. 2019; Bogaert and Gevaert 2020). Plant genomes encode hundreds of proteases, including metacaspases which are predicted to reveal potential destabilizing residues (Rawlings et al. 2018). Given the conditional nature of proteolytic cleavage, proteomic analyses and other strategies to identify substrates may need to compare N-degron pathway mutant alleles under different environmental conditions to reveal cryptic degrons and also incorporate subcellular fractionation to access low abundance targets. Cell and tissue specificity is also a key consideration for future studies.

#### Who regulates the regulators?

The Arg/N-degron pathways do not operate in isolation and a major outstanding question is how they intersect with other signaling pathways. This may be complex, for example, the activity of master regulator substrates such as ERFVIIs is subject to modulation by transcription factors, kinases, membrane association, degron masking, and additional E3 ligases (Licausi et al. 2011; Papdi et al. 2015; Lin et al. 2019; Liu et al. 2021; Fan et al. 2023). Furthermore, it is not yet fully understood to what extent different N-terminal modifications compete to influence protein fate in plants (Kats et al. 2018; Linster et al. 2022), and it remains to be explored whether plant Arg/N-degron pathways intersect with autophagic pathways as in mammals (Heo et al. 2023). Thus, whilst the importance of plant Arg/N-degron pathways in controlling the lifetime of regulatory proteins is well established, to what extent they contribute more widely to protein turnover and quality control remains an open question.

### What are the degrons and molecular players in the chloroplast N-degron pathway?

**(Written by Klaas J. Van Wijk)**

**Figure 6.**
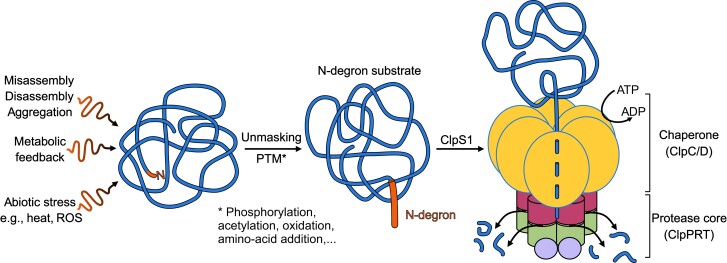
Schematic view of the N-degron pathway for degradation by the chloroplast Clp chaperone-protease system. Proteins can be converted into substrates for the Clp system by various events including protein complex disassembly and aggregation, different stresses such as heat and radical oxygen species (ROS), or through metabolic feedback (e.g. known to occur in the chlorophyll synthesis pathway). These changes to proteins can result in the generation of a degradation signal known as a degron, either by simply exposing (“unmasking”) the N-terminus of the protein or by a post-translational modification (PTM). Examples of such PTMs are phosphorylation, acetylation, oxidation, or the addition of an amino acid to the N-terminus. This N-degron is then recognized by the ClpS1 recognin (and possibly also ClpF), which delivers the bound substrate to the ClpC or ClpD chaperones for ATP-dependent unfolding and concomitant threading into the Clp protease complex. The unfolded substrates are degraded within the Clp proteolytic chamber resulting in the release of degradation products in the form of small peptides. However, the in vivo nature of these chloroplast N-degrons is yet to be determined. Elucidation of these N-degrons and the molecular players involved in their generation and recognition is a major challenge to be addressed.

N-degrons are major determinants of protein stability in the cytosol of bacteria and eukaryotes (Dissmeyer et al. 2018; Varshavsky 2019; Holdsworth et al. 2020; Weits et al. 2021) and likely also chloroplasts and non-photosynthetic plastids (Bouchnak and van Wijk 2019, 2021). Systematic mass spectrometry (MS) analysis of the N-termini of stromal-exposed proteins using N-terminal tagging (with a technique named TAILS; Rowland et al. 2015) showed enrichment of canonical stabilizing residues A, V, T, and S (often in N-α-acetylated form) and avoidance of charged (D, E, R, and K) and large hydrophobic residues (e.g. W, F, Y, and L) that serve as primary or secondary degrons in bacteria and eukaryotic cytosol (Rowland et al. 2015). We therefore postulated that an N-degron pathway exists in chloroplasts and other plastid types [Rowland et al. 2015; Bouchnak and van Wijk 2019 (Fig. 6)].

N-degron pathways in eukaryotes, including plants, typically involve polyubiquitylation and the proteasome (Perrar et al. 2019; Holdsworth et al. 2020; Weits et al. 2021). In contrast, the prokaryotic N-degron pathway depends on the adaptor ClpS (also named N-recognin) for the recognition and delivery of N-degron-bearing substrates to Clp chaperone-protease systems. The first step involves N-degron recognition of hydrophobic residues through a hydrophobic pocket in ClpS followed by docking of the ClpS-substrate complex on the N-domain of the ClpA or ClpC AAA+ chaperone (Kim et al. 2022). The ClpS-substrate complex is then “pulled” into the ClpA/C pore in an ATP-dependent fashion (requiring ATP hydrolysis), and the resulting distortion of the ClpS structure allows release of the substrate inside the ClpA/C pore. ClpS is subsequently released from ClpA/C, and the unfolding and degradation of the substrate by the Clp protease ring is completed (Kim et al. 2022).

Chloroplast ClpS1, a structural and functional homolog of bacterial ClpS, directly interacts with the ClpC chaperones (Nishimura et al. 2013; Nishimura and van Wijk 2015). ClpS1 affinity experiments in Arabidopsis identified several interacting chloroplast proteins, including glutamyl tRNA reductase 1 (GluTR) a key enzyme in tetrapyrrole biosynthesis (heme and chlorophyll) (Nishimura et al. 2013). Follow-up experiments showed that dark-induced degradation of GluTR indeed requires the Clp system (Apitz et al. 2016; Richter et al. 2019). The interaction between ClpS1 and these candidate substrates was dependent on the conserved substrate-binding residues in ClpS1 (Nishimura et al. 2013). However, N-degrons in these substrates have not been identified and no obvious canonical N-degrons were found. In vitro ClpS1 affinity assays with selected recombinant N-degron reporters demonstrated that ClpS1 has a restricted N-degron specificity (Montandon et al. 2019a). Furthermore, a high-resolution structure (2 Å) for Arabidopsis ClpS1 showed that the N-degron binding pocket of ClpS1 is slightly enlarged compared to that of *Escherichia coli* ClpS (Kim et al. 2021). In addition, amino acid replacement from Val (in *E. coli*) to Ala in ClpS1 caused a reduction in hydrophobic interactions with Leu N-degrons (Kim et al. 2021). Peptide array experiments with recombinant ClpS1 showed that N-terminal acetylation prevented the binding of such N-termini to ClpS1 (Aguilar Lucero et al. 2021). Collectively, these in vitro and in vivo data suggest a unique N-degron pathway in chloroplasts. Recent studies show that bacterial ClpS can also recognize non-canonical N-degrons including degrons a few residues downstream of the N-terminus (Gao et al. 2019a; Jin et al. 2021); hence this scenario should also be considered for chloroplasts.

ClpF was identified as an interactor of ClpS1 and it was shown that ClpF and ClpS1 mutually stimulate their association with ClpC in vivo (Nishimura et al. 2015). Identified interactions between ClpF, ClpS1, and GluTR suggested a ternary complex, and a testable model was proposed in which ClpS1 and ClpF form a binary adaptor for selective substrate recognition of GluTR (and perhaps other proteins) and delivery to ClpC (Nishimura et al. 2015). Whereas ClpF is a direct interactor to ClpS1 as well as ClpC1, the mechanistic role of ClpF in the N-degron pathway is not understood.

To identify additional Clp substrates, an in vivo ClpC1 substrate trap with a C-terminal STREPII affinity tag was expressed in Arabidopsis. This ClpC1-trap has mutated critical glutamate residues (E374A and E718A) in the two Walker B domains of ClpC1 required for ATP hydrolysis [ClpC1-TRAP (Montandon et al. 2019b; Rei Liao et al. 2022)]. Based on homology to non-plant ClpB/C chaperones, it is predicted that interacting substrates are not released; i.e. they are trapped (Rei Liao and van Wijk 2019). Affinity purification of the ClpC1-TRAP resulted in more than 50 highly enriched proteins compared to affinity-purified wild-type ClpC1 (Montandon et al. 2019b; Rei Liao et al. 2022). These included >20 small proteins with unknown function/domains and several metabolic enzymes some of which were also identified as ClpS1 interacting proteins or over-accumulated in *clp* mutants (Nishimura et al. 2013). These enriched proteins likely represent Clp protease substrates, some possibly with N-degrons, and/or new adaptors.

Despite the significant support for a unique N-degron pathway in chloroplasts that involves ClpS1, and perhaps also ClpF, in vivo demonstrations for ClpS1-dependent substrate selection and delivery to the Clp chaperone-protease system are lacking. For instance, whereas in vitro peptide binding assays for Arabidopsis ClpS1 showed a clear affinity for hydrophobic N-terminal amino acids (in particular, F, W, and Y), the ClpS1 protein interactome data with stromal proteins (e.g. for GluTR) failed to suggest a canonical N-degron. Furthermore, a direct positive correlation between chloroplast protein N-termini and ClpS1-dependent degradation has not been shown. Key questions about chloroplast N-degron pathways that need to be resolved include: (i) does generation of chloroplast N-degrons involve post-translational modifications (e.g. acetylation, phosphorylation, oxidation, or amino acid transfer), and are there specific enzymes involved in creating these modifications? (ii) are N-degrons for ClpS1 generated by N-terminally truncation of upstream proteases as has been suggested in *E. coli* (Humbard et al. 2013)? (iii) are N-degrons confined to the very N-terminal residue of the substrates or are there more downstream signals (non-canonical N-degrons)? (iv) is ClpS1 aided by co-adaptors such as ClpF? (iv) is there competition between ClpS1 and other adaptors/anti-adaptors—to influence substrate selection and regulation of rates of proteolysis? Novel and innovative in vivo chloroplast tools and approaches are needed to determine the molecular details of ClpS1-dependent mechanisms of N-degron substrate selection and delivery to the Clp chaperone-protease system in chloroplasts.

## Roles of proteolysis in developmental and metabolic signaling

### How do plants use ubiquitin-mediated proteolysis to regulate photomorphogenesis?

**(Written by Ning Wei and Giovanna Serino)**

**Figure 7.**
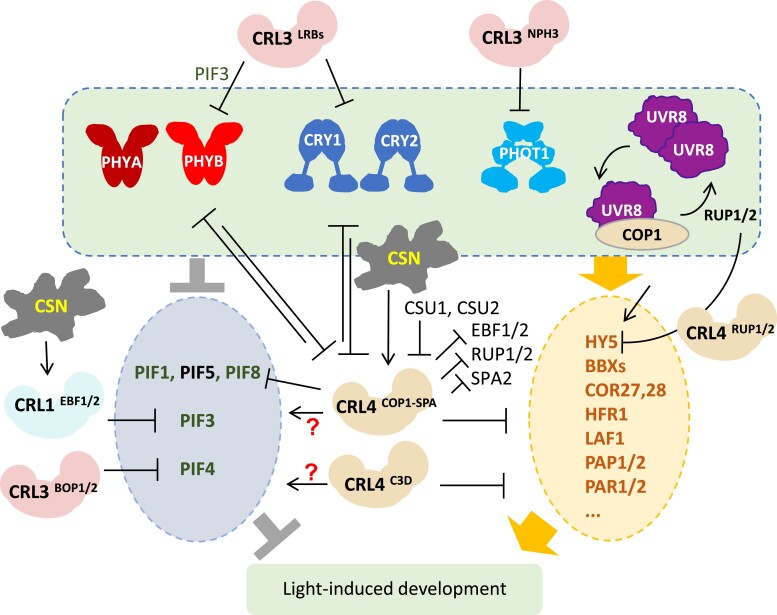
Regulation of light-signaling components through the UPS. Photoreceptors, various light-promoting transcription factors (inside the yellow circle on right), and light repressors (inside the blue circle on left) are regulated by UPS through indicated CRL E3 ligase complexes.

Light signals perceived by photoreceptors are transduced to guide plant growth and development in a process known as photomorphogenesis. Dark-grown etiolated seedlings undergo dramatic changes after exposure to light, including inhibition of hypocotyl elongation, unfolding of the apical hook, expansion of cotyledons, and maturation of chloroplasts (Kendrick and Kronenberg 1994). Genetic screens for Arabidopsis mutants exhibiting longer hypocotyls in the light led to the identification of photoreceptor mutants such as *long hypocotyl 3/phytochrome B* [*hy3*/*phyB*] and *long hypocotyl 4*/*cryptochrome 1* [*hy4/cry1*], and *hy5, a* mutant of a positive regulator of photomorphogenesis (Koornneef et al. 1980). Genetic screens for mutants with short hypocotyls and open cotyledons in the dark identified the *constitutively photomorphogenic/de-etiolated/fusca* (*cop/det/fus*) mutants (Chory et al. 1989; Deng et al. 1991; Wei and Deng 1992). As it turned out, all of the corresponding gene products function via the UPS, underscoring its importance in photomorphogenesis, as they encode components of E3 Ub ligase complexes CRL4^COP1-SPA^ (Ponnu and Hoecker 2021), CRL4^C3D^ (C3D: COP10-DDB1-DET1-DDA1), and of a CRL regulator, the COP9 Signalosome (CSN). The CSN complex regulates all CRLs by de-neddylation, i.e. removing the Nedd8/RUB1 modification of the cullin subunit (Schwechheimer et al. 2001; Qin et al. 2020). Mounting evidence shows that CRLs play key roles in photomorphogenesis, as they regulate the stability of many components of light signaling, from photoreceptors to transcription factors (Fig. 7).

#### Regulating photoreceptor stability

Photoactivated phyA (Seo et al. 2004; Saijo et al. 2008; Debrieux et al. 2013), phyB (Jang et al. 2010; Lu et al. 2015; Sheerin et al. 2015), and both CRYs (Chen et al. 2021c; Miao et al. 2022) are ubiquitylation targets COP1-SPA. In addition, under strong red light, phyB is recruited to the CUL3-based ligases CRL3^LRBs^ through LIGHT-RESPONSE BRIC-A-BRAC/TRAMTRACK/BROAD 1 AND LIGHT-RESPONSE BRIC-A-BRAC/TRAMTRACK/BROAD 2 (LRB1, LRB2) to be ubiquitylated (Christians et al. 2012; Ni et al. 2014). In the same fashion, CRL3^LRBs^ also mediate the degradation of cryptochrome 1 (CRY1) and CRY2 under high blue light or low temperature (Ma et al. 2021; Chen et al. 2021c; Miao et al. 2022). By lowering the level of phytochromes and cryptochromes, CRL3^LRBs^ serve to prevent over-stimulation and maintain light-signaling homeostasis. In addition, CRL3^NPH3^ targets protein degradation of the phototropin Phot1, a blue light-sensing photoreceptor mediating phototropic responses (Roberts et al. 2011; Fig. 7).

#### Regulating phytochrome interacting factor (PIF) stability

The stability of PIFs, which play essential roles in etiolation (skotomorphogenic development in darkness), as well as in shade avoidance and temperature responses under light conditions, is tightly controlled by the UPS. Light exposure results in rapid degradation of PIF proteins to induce de-etiolation. In this process, PIF3 is phosphorylated in response to photoactivation of phyB and then ubiquitylated by CRL1^EBF1/2^ for subsequent degradation (Dong et al. 2017). PIF3, together with phyB, is also a ubiquitylation target of CRL3^LRBs^ (Ni et al. 2014); however, this co-degradation occurs specifically under higher light intensity (Dong et al. 2017). Thus, the two different Ub ligases play opposite roles in phyB signaling in different light environments: CRL3^LRBs^ attenuate light signaling under high light irradiation, while CRL1^EBF1/2^ promotes photomorphogenesis, especially during de-etiolation.

PIF4 plays an important role in plant responses to shade, elevated temperature, and diurnal cycle. CRL3^BOP2^ has been shown to mediate PIF4 ubiquitylation and degradation, and indeed the *bop1 bop2* double mutant is hypersensitive to high temperature-mediated hypocotyl elongation (Zhang et al. 2017). In addition, PIF4 is phosphorylated by the brassinosteroid (BR) signaling kinase BRASSINOSTEROID-INSENSITIVE 2 (BIN2), resulting in its degradation during the diurnal cycle (Bernardo-García et al. 2014). It is probable that PIF4 is also modulated by other E3 ligases, yet to be identified, that are sensitive to PIF4 phosphorylation. Last but not least, light-induced degradation of PIF1 (Zhu et al. 2015), PIF5 (Pham et al. 2018), and PIF8 (Oh et al. 2020a) has been shown to involve the CRL4^COP1-SPA^ complex, arguing for a dual role of COP1 in light signaling.

#### Open questions: how does COP1 achieve multifaceted roles within the complexity of light signaling?

There are several open questions as to how UPS regulates light signaling. For example, specific E3s regulating PIF stability in a time-, space- and signal-dependent manner are still to be identified. Here, we focus on mechanisms centered around COP1 and its dual role in light signaling. The COP1-SPA complex acts as a central photomorphogenic suppressor by targeting myriad positive regulators of light signaling, such as HY5, LONG HYPOCOTYL IN FAR-RED LIGHT1 (HFR1), B-BOX DOMAIN PROTEIN4 (BBX4), photoreceptors, and many more, in dark or dim light conditions (Ponnu and Hoecker 2021; Fig. 7). However, some of these ubiquitylation targets are also stabilized by COP1 under different conditions. For example, while COP1 targets HY5 degradation in the dark, it stabilizes it during UV-B-mediated photomorphogenesis (Oravecz et al. 2006). While COP1 (as well as DET1) stabilizes PIFs in darkness to ensure etiolated development (Bauer et al. 2004; Dong et al. 2014; Gangappa and Kumar 2017; Ling et al. 2017), it also facilitates de-etiolation through CRL4^COP1-SPA^-mediated light-induced degradation of several PIFs (see above). Indeed, *cop1* seedlings not only have reduced PIF levels in darkness, but they also show defects in light-induced PIF degradation (Zhu et al. 2015; Pham et al. 2018).

Studies from the last 10 years have also revealed that light signals inactivate COP1 by altering the composition of COP1-associated complexes. UV-B irradiation causes dissociation of COP1-SPA from the CRL4 core complex (Huang et al. 2013) and the subsequent formation of a COP1-UVR8 complex (Rizzini et al. 2011; Wang et al. 2022). The binding of monomeric UVR8 to the VP substrate–recognition interface of COP1 dislodges COP1 substrates such as HY5, allowing them to accumulate and thus stimulating downstream light responses (Huang et al. 2014; Lau et al. 2019; Wang et al. 2022). Likewise, photoactivated phytochromes and cryptochromes promote the physical separation of COP1 and SPA proteins (Lian et al. 2011; Zuo et al. 2011; Lu et al. 2015; Sheerin et al. 2015; Ponnu et al. 2019). Thus, disassembly of the CRL4^COP1-SPA^ complex and blocking of the COP1 substrate-binding sites seem to be a general strategy in the light-dependent switch of COP1 activity. Similar to those photoreceptor-directed actions, CSU2 suppresses COP1 activity also by binding to the COP1 coiled-coil domain, which interferes with COP1 dimerization and the assembly of COP1-SPA complexes (Xu et al. 2015a).

However, how COP1 and DET1 stabilize PIFs in the dark remains an outstanding open question (Bauer et al. 2004; Dong et al. 2014; Gangappa and Kumar 2017; Ling et al. 2017). In the absence of COP1 or DET1, PIFs cannot accumulate. Since COP1 has been shown to target EBF1/2 to degradation (Shi et al. 2016), the F-box proteins that mediate ubiquitylation of PIF3 and EIN3 via CRL1^EBF1/2^ ligase, it is possible that *cop1* mutant may accumulate EBF1/2, which would lead to PIF3 destabilization. Additional mechanisms likely exist, and more rigorous investigations are needed to elucidate how COP1 and DET1 stabilize PIFs.

On the other hand, while it is clear that COP1-SPA serves as a substrate-recognition component in CRL4 ^COP1-SPA^, it remains obscure whether it can function as a stand-alone E3 ligase through the COP1 RING domain. COP1 function also requires CSN and DET1 (Qin et al. 2020; Cañibano et al. 2021), but the physical and functional interactions between COP1- and DET1-associated complexes require further clarification. The CSN complex has pleiotropic functions beyond light signaling, as it regulates most CRL ligases. In light signaling, CSN-mediated de-neddylation of CUL1 is necessary for loading the PIF3-EBF1 complex onto the CRL1 ligase, thus assembling the CRL1^EBF1^ holocomplex during light-induced PIF3 degradation (Dong et al. 2024) (Fig. 7). Dissecting the functions and dynamic interactions of COP1-, DET1-, and CSN-associated complexes remain highly challenging in the coming years.

### How does proteolysis of core signaling components occur in different subcellular locations to modulate the ABA pathway?

**(Written by Pedro L. Rodriguez)**

ABA is perceived by a family of ABA receptors known as PYRABACTIN RESISTANCE 1 (PYR1)/PYR1-like (PYL)/regulatory components of ABA receptors (RCAR), which leads to inhibition of clade A protein phosphatases type-2C (PP2Cs) and subsequent relief of inhibition of subfamily III Snf1-related protein kinases 2 (SnRK2s; Cutler et al. 2010). Additionally, B2/B3-type RAF-like MAP3Ks are required to phosphorylate and activate the above SnRK2s (Lin et al. 2021). Proteolytic targeting mediated by E3 Ub ligases is a central component of all phytohormone signaling pathways, including ABA (Blázquez et al. 2020). Pioneering work on E3 ligases that regulate protein levels of transcription factors associated with ABA signaling, such as ABSCISIC ACID INSENSITIVE3 (ABI3) and ABI5, established a link between the UPS and modulation of ABA signaling (reviewed by Stone 2019).

**Figure 8.**
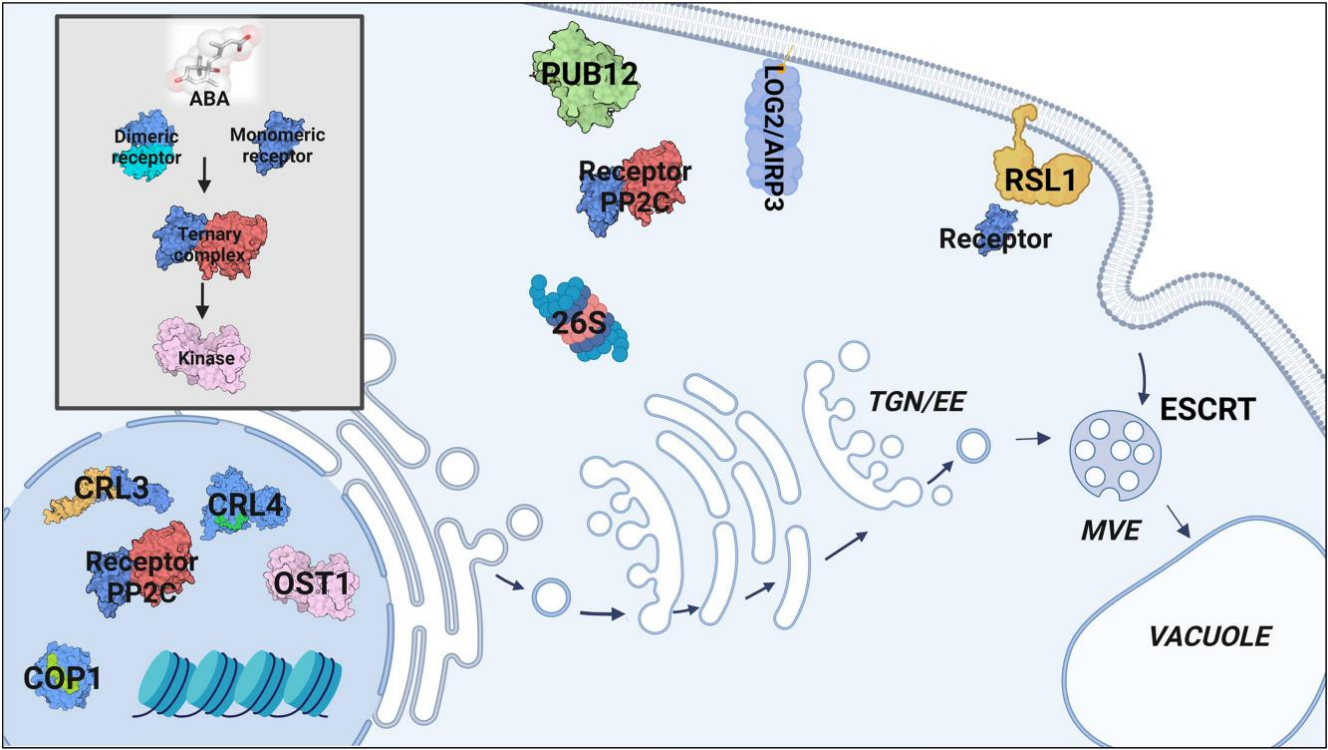
Proteolysis of core ABA signaling components occurs in different subcellular compartments. The inset shows that ABA is perceived through dimeric or monomeric receptors (blue), which triggers the formation of ternary complexes with clade A PP2Cs (red), and relief of inhibition of SnRK2.2/2.3/2.6 (pink) kinase activity. Nuclear, cytosolic, and PM targeting pathways of core components are indicated. RSL1 illustrates the targeting of ABA receptors at the PM, which promotes endosome-mediated vacuolar degradation via the ESCRT machinery, whereas PUB12 and AIRP3 might target PP2Cs in the proximity of the PM and follow either cytosolic or vacuolar degradation pathways. Nuclear degradation of ABA receptors, PP2Cs, and OST1 involves the multimeric CRL3, CRL4, and RING-type COP1 E3 ligases, among others (see text for details). Nuclear and cytosolic 26S proteasomes and the vacuole participate in the degradation of core signaling components, which might influence signaling, desensitization, or resetting of the ABA pathway. The lytic vacuole also receives cargo for degradation via autophagy but data linking ABA with autophagy are limited. The figure was created using BioRender (https://biorender.com).

Further work served to identify E3 ligases that regulate protein levels of other core components of ABA signaling, i.e. ABA receptors, PP2Cs, and SnRK2s (reviewed by Ali et al. 2020; Coego et al. 2021). ABA signaling in Arabidopsis involves 14 ABA receptors and 9 PP2Cs, as well as 3 SnRK2s. This is mirrored in the high number of E3 ligases that target these proteins (Ali et al. 2020; Coego et al. 2021). Because these E3 ligases are located in different cell compartments, the connection between plant cell biology and the regulation of ABA signaling is an emerging question for research (Fig. 8). Processing at the plasma membrane (PM) leads to cargo degradation into the lytic vacuole, via the endocytic pathway and autophagy (Saeed et al. 2023). Moreover, PM signaling nanodomains might be physiologically connected with the endocytic pathway and E3 ligases targeting ABA signaling components (Yu and Xie 2017; Chen et al. 2023c). The RBR-type E3 ligase RSL1 targets ABA receptors in the PM and promotes their endosome-mediated vacuolar degradation (Bueso et al. 2014). Given that ABA signaling at the PM is critical for the regulation of ion and water transporters, E3 ligases anchored in the PM through transmembrane domains, myristoylation, or as peripheral proteins might contribute to K63Ub-mediated targeting of core signaling components to the endovacuolar pathway (Bueso et al. 2014; Belda-Palazon et al. 2019; Pan et al. 2020). K63-linked Ub chains not only act as a signal for endocytosis but also might contribute to the autophagic clearance of cargo proteins that act in ABA signaling (Sirko et al. 2021; Saeed et al. 2023).

Proteolytic targeting of all the players in ABA signaling, including ABA receptors, PP2Cs, SnRK2s, and TFs in the nucleus, has been reported, affecting ABA transcriptional regulation and likely long-term ABA-induced changes in chromatin arrangement. Multimeric CRL4 E3 ligases regulate ABA receptor and OST1 protein levels in the nucleus through different substrate adaptor modules, involving DDA1 and HOS15, respectively (Irigoyen et al. 2014; Ali et al. 2019). On the other hand, the CRL3^BPM^ or the RING-type COP1 E3 ligases regulate nuclear PP2C protein levels (COP1 also in the cytosol) and affect stomatal function (Julian et al. 2019; Chen et al. 2021a). ABA induces chromatin remodeling in many cell types, affecting, for example, the root, the guard cell, and the mesophyll cell epigenome (Seller and Schroeder 2023). SnRK2s and PP2Cs orchestrate a phosphorylation-based switch to control the SWI/SNF chromatin-remodeling ATPase BRAHMA activity, which might be sensitive to developmental and environmental signals that regulate their protein levels (Peirats-Llobet et al. 2016). A possible memory effect of proteolysis on chromatin remodeling in response to abiotic stress deserves further investigation.

Other E3 ligases that target PP2Cs are located near or associated with the PM, such as PUB12/13 and LOG2/AIRP3 specifically targeting ABI1, or RGLG1/5 for PP2CA (Kong et al. 2015; Wu et al. 2016; Pan et al. 2020). Only in the case of RGLG1 has the subcellular localization of the PP2CA-RLG1 interaction been investigated, and interestingly, it was found that ABA modifies the PM localization of RGLG1 and promotes nuclear interaction with PP2CA (Belda-Palazon et al. 2019). ABA enhances the interaction of the E3 ligase and its target, and elucidation of this ABA-dependent translocation represents an area for further research. In the case of PUB12/13, the ubiquitylation of ABI1 in vitro requires exogenous ABA and the presence of ABA receptors. This suggests that some E3 ligases can recognize the PP2C-Receptor complexes, whose formation requires ABA for the dissociation of dimeric receptors and the assembly of highly stable forms (both for dimeric and for monomeric receptors). This model also applies to RGLG1, which forms nuclear complexes with PP2CA and monomeric receptors, such as RGLG1-PP2CA-PYL8 (Belda-Palazon et al. 2019).

However, in another example, ABA protects the PYL8-ABA receptor from degradation (Irigoyen et al. 2014). Thus, when ABA levels increase, the CRL4^DDA1^ complex cannot promote the degradation of PYL8, establishing a positive feedback loop for PYL8-dependent signaling. It is not known whether the assembly of the CRL4^DDA1^ complex is impaired by ABA or if PYL8-ABA-PP2C complexes are resistant to degradation by CRL4^DDA1^. Finally, the activation of SnRK2 degradation by ABA is postulated to be a negative feedback loop when SnRK2s are phosphorylated by B2/B3-type RAF kinases (Lin et al. 2021), as well as the post-translational modification of ABA receptors can accelerate receptor degradation (Castillo et al. 2015; Yu et al. 2019). This suggests that some E3 ligases might be sensitive to post-translational modifications of their targets, but the precise mechanism is unknown.

In summary, the interaction of different E3 ligases with core components of ABA signaling has been reported in the PM, cytosol, and nucleus, and additionally, the endosomal trafficking pathway plays a key role in the turnover of ABA receptors that have been ubiquitylated at the PM (Belda-Palazon et al. 2016; Yu et al. 2016; Garcia-Leon et al. 2019). ABA plays signaling roles, or its levels are increased, in other subcellular compartments in response to abiotic stress, such as mitochondria or endoplasmic reticulum (Han et al. 2020; Postiglione and Muday 2023). This suggests that ABA perception and regulation of the half-life of certain core signaling components might occur in particular cell regions, and regulation of the local concentration of the core signaling network might be achieved by yet-to-be-discovered E3 ligases. The degradation of key repressors of the ABA pathway (i.e. PP2Cs) is not the only mechanism to activate signaling because of the alternative biochemical inhibition of their activity by ABA receptors (Cutler et al. 2010). This poses unique questions to fully understand ABA signaling open for further research. For example, how is biochemical (reversible) inhibition of PP2Cs intertwined with their proteolytic degradation, either when they are free or in ternary complexes with ABA and ABA receptors? And from a global perspective, is proteolytic degradation involved in signaling, desensitization, or resetting of the ABA pathway?

### The complexity of the strigolactone signaling pathway: how does the D14 receptor function as both receptor and enzyme, linking hormone perception to protein degradation?

**(Written by Angelica M. Guercio, Malathy Palayam, and Nitzan Shabek)**

Strigolactones (SLs), initially identified as root exudates from cotton [*Gossypium hirsutum* (Cook et al. 1966)], were first described to have a role in hormone signaling in the control of shoot branching (Gomez-Roldan et al. 2008; Umehara et al. 2008). Over the years, SLs have been further characterized, and the cohort of diverse processes controlled by SLs is still expanding. SLs can impact plant-environment interactions such as initiating symbiosis with mycorrhizal fungi and stimulating the germination of parasitic plants (Bouwmeester et al. 2003; Akiyama et al. 2005, 2010; Yoneyama et al. 2010; Gutjahr et al. 2015). Endogenously, SLs regulate various aspects of plant growth and development, including shoot branching, leaf growth, leaf senescence, secondary stem thickening, the formation of adventitious roots, lateral roots, and root hairs (Brewer Koltai and Beveridge 2013; Ruyter-Spira et al. 2013; Bennett and Leyser 2014; Smith and Li 2014). Research on the extensive crosstalk between SL signaling and other phytohormones continues to unveil a comprehensive network of cross-hormone regulation and diverse effects in plant physiology.

#### The crossroads of plant hormones and the Ub system

Similar to other hormone signaling pathways in plants, SL signaling relies on regulated turnover through the UPS (Jiang et al. 2013; Zhou et al. 2013a; Tal et al. 2020). Before the identification of the SL receptor, the F-box protein MORE AXILLARY GROWTH 2 (MAX2) or DWARF3 (D3) in rice, a component of the SCF (Skp1/Ask1- Cullin1-F-box) type E3 Ub ligase, was recognized as a key player in SL-related pathways (Stirnberg et al. 2007). *max2* mutant plants exhibited phenotypes similar to those with mutations in SL biosynthesis pathways. However, unlike other SL mutants, *max2* phenotypes could not be rescued with SL treatment, indicating the involvement of MAX2 in SL signaling. Later, the SL receptor was discovered through mutant analysis (Arite et al. 2009), and similar to GA signaling, the receptor is an α/β hydrolase commonly referred to as D14 (DWARF14). It was hypothesized that D14, bound to SL, initiates complex formation with the SCF^MAX2/D3^ which subsequently recruits and orchestrates the polyubiquitylation and proteasomal degradation of the target proteins such as D53 (DWARF53 in rice) or SMXL6/7/8 (SUPPRESSOR OF MAX2 LIKE 6, 7, and 8) (Jiang et al. 2013; Zhou et al. 2013a; Bennett and Leyser 2014; Wang et al. 2015). D53/SMXLs have a weak homology with class I Clp ATPase family proteins and have been shown to regulate SL response genes via their EAR motifs, but their precise functions and transcriptional targets remain elusive (Jiang et al. 2013).

#### Decoding the dual role of the SL receptor D14

The SL receptor D14 has a dual role as both a receptor and an enzyme, capable of hydrolyzing SL (Hamiaux et al. 2012). Structural biology has played a vital role in unraveling the intricate mechanisms behind SL perception. Crystal structures of D14 from various plant species have shown a common α/β hydrolase fold with a deep ligand-binding pocket formed by a V-shaped lid comprised of four α-helices (Hamiaux et al. 2012; Kagiyama et al. 2013; Guercio et al. 2023). The bottom of the ligand-binding pocket contains a conserved serine catalytic triad, highlighting the hydrolase activity that has been preserved throughout plant evolution (Bythell-Douglas et al. 2017). Studies have further examined the evolutionary history of these receptors, and the co-evolution of the residues lining the ligand-binding pocket to provide specificity for diverse SL molecules (Guercio et al. 2023).

Yet, an open question centers on the necessity of SL hydrolysis for propagating SL signaling. While it was initially proposed that hydrolysis induced conformational changes in D14, allowing it to interface with D3 (Yao et al. 2016), recent research has challenged this view. Mutants of D14 incapable of catalyzing SL hydrolysis were shown to bind SL and rescue *d14* mutant phenotypes in an SL-dependent manner, indicating that SL binding, rather than hydrolysis, can initiate the signaling cascade (Seto et al. 2019). The different states of intact SL binding and SL hydrolysis may convey distinct signals, with MAX2 directing the rate of SL hydrolysis, interactions, and the proteasomal degradation of D14 and SMXLs (Shabek et al. 2018; Marzec and Brewer 2019).

#### Mounting complexity: dynamics of E3 Ub ligase influence substrate degradation

Evidence to date demonstrates that D14 binding to MAX2/D3 is required to mediate the degradation of the target substrate. However, the reported crystal structures containing the D14–D3 complex introduce additional complexity. One such structure reveals a significant conformational change in D14 when complexed with D3, presumably in a post-SL hydrolysis state (Yao et al. 2016). This observation is supported by the identification of a covalently linked intermediate molecule (CLIM) formed during SL hydrolysis, indicating that hydrolysis and conformational change play roles in D14–SL–D3 complex formation.

**Figure 9.**
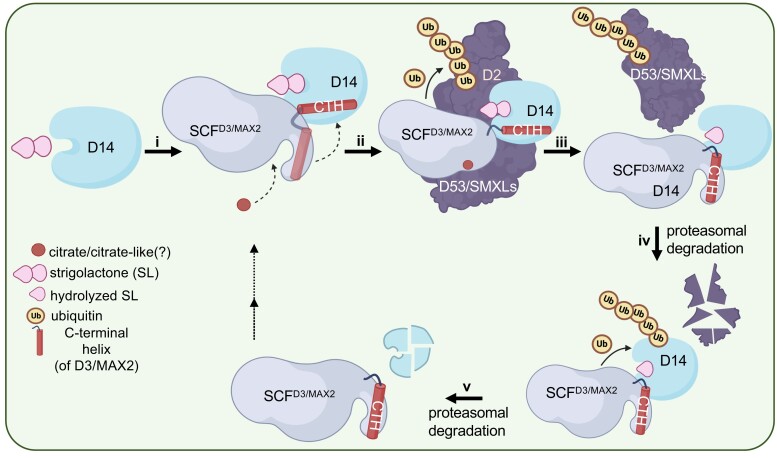
Regulation of SL signaling through protein degradation: (i) SL (light pink) is perceived by the receptor D14 (blue). (ii) The activated SL receptor then binds to D3/MAX2 F-box (gray) as part of the SCF^D3/MAX2^ complex. The presence of citrate or citrate-like molecules (red) triggers a conformational change in the C-terminal helix, CTH (red) of D3/MAX2 (gray), causing it to dislodge. (iii) The dislodged CTH of D3/MAX2-D14 complex subsequently loads the transcriptional repressor, D53/SMXLs (purple), through its D2 domain leading to polyubiquitylation (Ub, yellow). The binding of D53/SMXLs reactivates the hydrolysis of SL by D14 either during or after the polyubiquitylation of D53/SMXLs. (iv) The hydrolysis of SLs triggers a conformational change of D14 and restores the CTH of D3/MAX2 to its engaged conformation and subsequently triggers the release of polyubiquitylated D53/SMXLs from the D14-D3/MAX2 to proteasomal degradation. (v) D14 undergoes ubiquitylation and proteasomal degradation completing the feedback regulation of the SL signaling cascade. Interestingly, before D53/SMXLs are released to the 26S proteasome, their transient interaction with D3 alters D14 inhibition and gradually restores SL hydrolysis (Shabek et al. 2018; Tal et al. 2022). This restored activity can effectively “reset” the SL signal by depleting the hormone and degrading the D14 receptor until the next cue. This E3 ligase domain plasticity provides an additional level of signaling control and represents a unique mode of targeting substrates for proteasomal degradation in the realm of phytohormone and UPS signaling. The figure was created using BioRender (https://biorender.com).

Another crystal structure unveils a stable form of D14 in complex with the C-terminal helix (CTH) of D3. Furthermore, it was demonstrated that D3 exists in multiple functional conformations, characterized by a flexible, highly conserved CTH (Shabek et al. 2018). This CTH plays an essential role in directly binding D14–SL as a dislodged form, leading to allosteric inhibition of SL hydrolysis. The dislodged state of MAX2/D3 can be triggered by a small molecule like citrate by binding to the D-pocket of the MAX2/D3 protein, displacing the C-terminus D720 and inducing a conformational switch. This regulatory action of citrate is crucial for modulating the spatial arrangement of the D14–D3 complex during SL perception and hydrolysis, as evidenced by augmented inhibition of SL hydrolysis by D14–D3 when treated with citrate (Tal et al. 2022). The D3/MAX2 open conformation provides a new interface for the recruitment of D53/SMXLs and their subsequent ubiquitination and degradation by SCF^MAX2/D3^ (Fig. 9). Despite its weak binding affinity, citrate's regulatory role is significant due to its dynamic cellular concentrations, influenced by environmental cues, and its potential impact on the fine-tuning of plant developmental processes and responses to stress.

The link between endogenous SL allocations, phosphate-poor soils, and the overproduction of citrate suggests a complex interplay between SL signaling and organic acid metabolism (López-Bucio et al. 2000; Brewer et al. 2013; Saeed et al. 2017; Liu et al. 2018a; Tahjib-Ul-Arif et al. 2021). Moreover, domain analysis of D53 illustrates four major domains (N, D1, M, and D2 domains) also featured by other AAA+ ATPase family members. It was revealed that the rice D53 D2 domain independently establishes a stable complex with D3–D14 and undergoes degradation through the UPS, suggesting that the D2 domain alone is competent for hormone-induced protein turnover catalyzed by D14-SL-SCF^MAX2/D3^ complex (Shabek et al. 2018). Subsequently, other studies have utilized the D2 domain as the best proxy to follow SL signaling and D53/SMXL-dependent degradation, in a cell-free system, in planta, or as a Strigo-D2 biosensor (Shabek et al. 2018; Song et al. 2021; Tal et al. 2022).

#### Outlook: receptor turnover and SL catabolism tug of war

The SL receptor D14 undergoes ubiquitylation and degradation by the 26S proteasome in an SL-dependent manner, creating a negative feedback mechanism (Chevalier et al. 2014; Hu et al. 2017). SL induces the rapid degradation of D53 within a few minutes, subsequently regulating the expression of SL-responsive genes while elevating D53 expression after about 1 to 2 h (Hu et al. 2017). However, the SL-induced degradation of D14 begins approximately 1 h after exposure and reaches its peak at around 3 to 4 h, indicating that precise feedback loops operating at different time intervals effectively modulate the duration and strength of SL signaling (Fig. 9). While further studies are needed to elucidate the precise mode of action of D14 degradation, it is hypothesized that upon SL hydrolysis, structural changes of the D14 fold occurs while bound to MAX2/D3, enabling the mediation of ubiquitylation and degradation of D14 (Chevalier et al. 2014; Hu et al. 2017; Shabek et al. 2018; Tal et al. 2022). The control of D14 levels and, subsequently, cellular SL levels hold promise as an intriguing new area of research to better understand the interplay between SL signaling and the UPS.

While D14 can hydrolyze SLs, it acts as a relatively slow enzyme in the regulation of SL depletion and/or inactivation. Recently, carboxylesterase CXE15 and CXE20 were found in Arabidopsis to effectively deplete SL levels (Roesler et al. 2021; Xu et al. 2021). Therefore, it is possible that the role of D14 in breaking down SLs is insignificant compared to carboxylesterase activity, and the SL hydrolysis process may solely aim to precisely tune up distinct conformational states of the enzyme and contribute to the regulation of the SL complex.

In conclusion, the perception and signaling cascade of strigolactone is characterized by its dynamic and complex nature, differing molecularly from other well-characterized phytohormone signaling pathways. The multifaceted processes governing strigolactone perception and signaling regulation through proteasomal degradation represent another evolving phytohormone field in the intricate world of plant hormones.

### Who takes the lead in the intricate dance between autophagy and sugar metabolism?

**(Written by Tamar Avin-Wittenberg)**

Autophagy is a vital mechanism for recycling nutrients, mitigating the impact of starvation (Takeshige et al. 1992; Meijer and Codogno 2004). The initial studies characterizing autophagy-deficient (*atg*) Arabidopsis mutants underscored their sensitivity to carbon and nitrogen starvation (Doelling et al. 2002; Hanaoka et al. 2002). Despite the connection between cellular carbon status and autophagy and the reciprocal influence of autophagy on metabolite availability, the interplay between carbon-containing metabolites, particularly sugars, and autophagy remains unclear. I will outline the known factors, persisting uncertainties, and challenges in exploring this issue.

**Figure 10.**
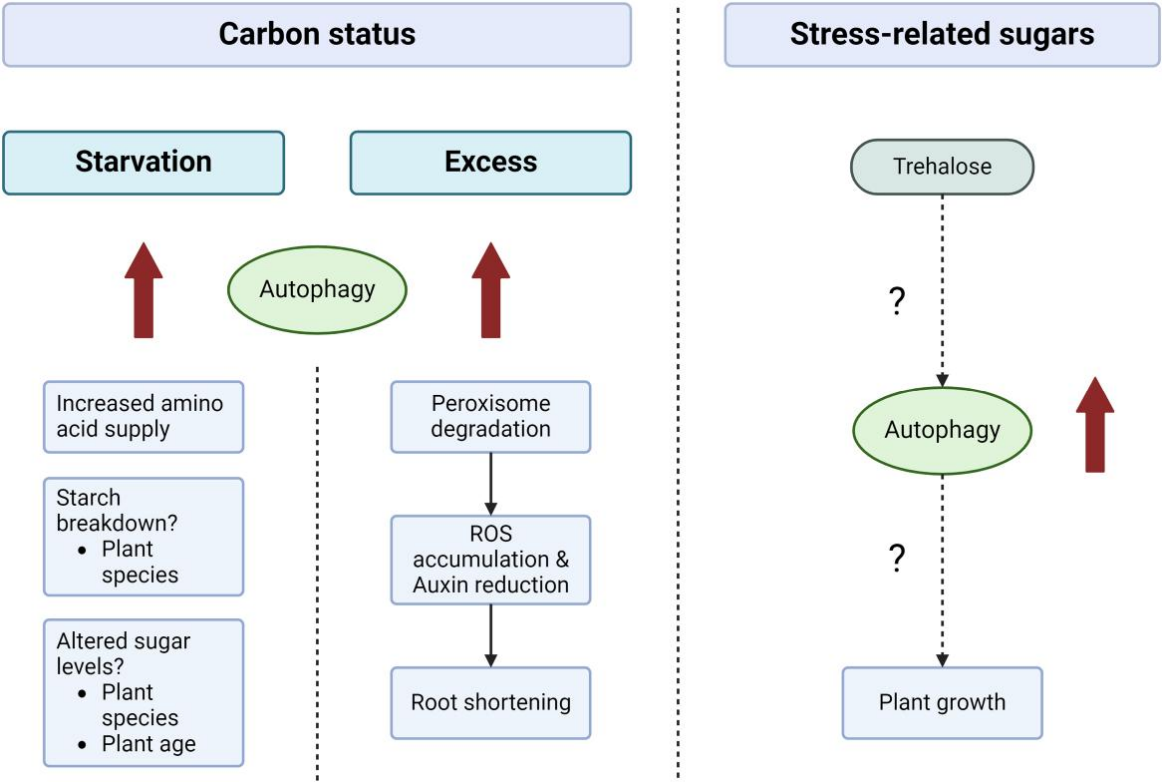
Interplay between carbon-containing metabolites and autophagy. The figure was created using BioRender (https://biorender.com).

Several groups have conducted metabolic profiling of *atg* mutant plants under both favorable and starvation conditions (Izumi et al. 2013; Masclaux-Daubresse et al. 2014; Avin-Wittenberg et al. 2015; Barros et al. 2017; McLoughlin et al. 2018; McLoughlin et al. 2020). Most studies reported changes in amino acid levels, aligning with autophagy’s role as a protein degradation mechanism (Fig. 10). However, some studies also observed alterations in sugar levels. For instance, slight sucrose accumulation was observed in Arabidopsis and maize (*Zea mays*) *atg* mutants under favorable conditions (Masclaux-Daubresse et al. 2014; Barros et al. 2017; McLoughlin et al. 2018). Additionally, Raffinose family oligosaccharides (galactinol, raffinose, and stachiose) accumulated in *atg* mutants under favorable and carbon starvation conditions (Masclaux-Daubresse et al. 2014; Avin-Wittenberg et al. 2015; McLoughlin et al. 2018).

Defining a consistent “sugar fingerprint” for autophagy deficiency proves challenging, as many of the changes in sugar levels are experiment-specific. Starch is also important in the context of carbon supply, as autophagy was suggested to function in starch breakdown in Arabidopsis (Wang et al. 2013b), and a cross between starchless and *atg* mutants increased cell death under short-day conditions (Izumi et al. 2013). Interestingly, maize *atg12* mutants demonstrated increased starch breakdown under carbon starvation (McLoughlin et al. 2020), while Arabidopsis *atg* mutants exhibited starch accumulation under carbon starvation (Barros et al. 2017), suggesting that autophagy-starch regulation is more complex (Fig. 10).

Several factors could explain the variations in sugar phenotypes observed in *atg* mutant experiments. First, two plant species were analyzed in the aforementioned studies, and the differences might point to species-specific roles of autophagy. As the study of plant autophagy expands to more species, additional data may reveal common factors in autophagy and metabolism. Secondly, the variability in Arabidopsis plant age during experiments adds complexity, potentially influencing metabolomics results. The variability may imply that autophagy could assume distinct roles at different stages of plant development, influencing sugar metabolism. Finally, the setup of carbon starvation may trigger a differential metabolic response. Previous studies have demonstrated that individual-leaf darkening induces leaf senescence, while whole-plant darkening inhibits it (Weaver and Amasino 2001). Moreover, a different metabolic response has been observed in both scenarios (Law et al. 2018). It is plausible that autophagy operates differently under these carbon starvation conditions, resulting in diverse metabolic outcomes (Fig. 10).

Several studies investigated autophagy and sugar excess. Arabidopsis *atg* mutant seedlings are less sensitive to elevated glucose and sucrose, but not fructose levels, displaying reduced inhibition of root growth upon high sugar treatment (Huang et al. 2018; Laloum et al. 2022). Surprisingly, this reduced inhibition of root growth is not attributed to changes in sugar metabolism or accumulation. Instead, it is connected to reduced reactive oxygen species (ROS) accumulation in the roots and the persistence of auxin levels in *atg* mutants, possibly due to reduced pexophagy, allowing for reduced inhibition of root growth [Fig. 10 (Huang et al. 2018)].

There is another point to consider when studying metabolism in knockout mutants. The metabolic phenotype may not arise solely from the absence of autophagy but could also stem from the pleiotropic effects of the mutation. Additionally, the role of autophagy in nutrient remobilization from source to sink tissues adds further complexity to the analysis. For instance, sucrose accumulation was observed in *atg12* mutant maize seeds (Barros et al. 2023) and *ATG4-RNAi* tomato (*Solanum lycopersicum*) fruit (Alseekh et al. 2022). However, whether this accumulation is a consequence of autophagy deficiency in the source or sink tissue remains uncertain. Reciprocal crosses between wild-type and *atg* mutant Arabidopsis plants did not reveal significant differences in sugar and lipid levels (Erlichman et al. 2023). This suggests that autophagy primarily functions in nitrogen remobilization rather than carbon remobilization. Thus, it is speculated that sucrose accumulation may result from localized autophagy effects rather than carbon remobilization from the source.

How are sugar levels involved in the regulation of autophagy? Two key kinase complexes, target of rapamycin (TOR) and Snf1-related protein kinase 1 (SnRK1), play a crucial role in nutrient sensing (Janse van Rensburg et al. 2019). TOR promotes plant growth and inhibits autophagy, while SnRK1 induces autophagy in response to starvation. SnRK1 activation of autophagy can occur either through TOR inhibition or direct activation. TOR is activated by various signals, including cellular glucose levels, though the specific details of this activation remain unknown (Mugume et al. 2020). SnRK1 senses energy and nutrient levels through adenine nucleotides (ATP, ADP, or AMP) or sugar phosphates. Trehalose 6-phosphate (T6P) acts as a negative regulator of SnRK1, representing cellular sucrose levels (Jamsheer et al. 2021). Recent research also revealed a connection between three glycolytic enzymes and autophagy inhibition. These enzymes bind to ATG101, a regulatory subunit of the kinase ATG1, to restrict its activity (Lee et al. 2023).

Recent findings indicate that sugars can induce autophagy. The stress-related sugar trehalose accumulates in Arabidopsis during carbon starvation (Barros et al. 2017), and its buildup during desiccation in the resurrection plant *Tripogon loliiformis* led to autophagy activation (Williams et al. 2015). Additionally, inhibiting trehalose degradation in maize activated autophagy and enhanced plant biomass [Sun et al. 2022 (Fig. 10)]. These results align with a growing body of evidence linking autophagy enhancement with increased plant performance (Minina et al. 2018).

In summary, the complex relationship between autophagy and sugar metabolism poses challenges in distinguishing cause and effect. The development of innovative tools, including inducible mutant lines and tissue-specific downregulation, is essential to mitigate the pleiotropic effects of knockout mutants. Furthermore, uncovering novel sugars that regulate autophagy and understanding their mode of action will contribute to a better understanding of this elaborate co-regulation.

### What is the role of proteolysis in fruit ripening regulation?

**(Written by Sergey Mursalimov and Simon Michaeli)**

Humans and other animals benefit from the ability of plant organs to survive detached while temporarily maintaining their taste, scent, and nutritional values. However, the lifespan of harvested leaves, roots, tubers, and fruits is limited without the continued supply of water and nutrients. Hence, their existence is, by default, subject to stressful conditions of energetic deprivation and dehydration. Moreover, due to trade and consumer requirements, fresh plant produce will experience extreme temperature and humidity fluctuations and be subjected to mechanical damage (Pedreschi and Lurie 2015; Al-Dairi et al. 2022). The latter further accelerates the appearance of biotic stressors, mostly fungi (Prusky and Romanazzi 2023). Changes in oxygen and CO_2_ levels during storage can affect respiration and also lead to oxidative damage (Pedreschi and Lurie 2015). These stressors may trigger protein misfolding and aggregation (Liu and Howell 2016), negatively affecting fresh produce's quality and shelf-life. Food loss and waste is estimated at around 40% worldwide (Porat et al. 2018) and is accompanied by economic damage and carbon footprint. Therefore, increasing plant-based food security requires both increasing crop yields and decreasing food loss waste (Foley et al. 2011).

Proteolysis pathways are pivotal in plant development, senescence, and stress responses and, as such, may prove to be efficient targets for plant-based reductions in food loss waste. The postharvest research field is vast. Here, I will focus on climacteric fruit ripening. Climacteric fruits, such as bananas, mangos, apples, and tomatoes, can ripen postharvest, and their ripening is accompanied by respiration and ethylene bursts (Cherian et al. 2014).

#### The UPS in ripening

The UPS is essential in ripening, primarily due to its involvement in the tight regulation of phytohormones, including auxin, ABA, and ethylene (Fenn and Giovannoni 2021). Notably, UPS components identified as hormone modulators in Arabidopsis were later found necessary in fruit ripening. For example, Ethylene-Insensitive 3 (EIN3)-Binding F-box (EBF) proteins are known to target EIN3, a key transcription factor in ethylene signaling, for proteasomal degradation (Guo and Ecker 2003; Potuschak et al. 2003). More recently, the role of EBFs in tomato and pear ripening (Yang et al. 2010; Deng et al. 2018; Wang et al. 2023a) and carnation petal senescence (Zhu et al. 2023) was demonstrated. The UPS may also regulate fruit traits independently of hormonal crosstalk. Fruit color has ecological and postharvest implications, affecting animal-borne seed dispersal and retail consumption.

**Figure 11.**
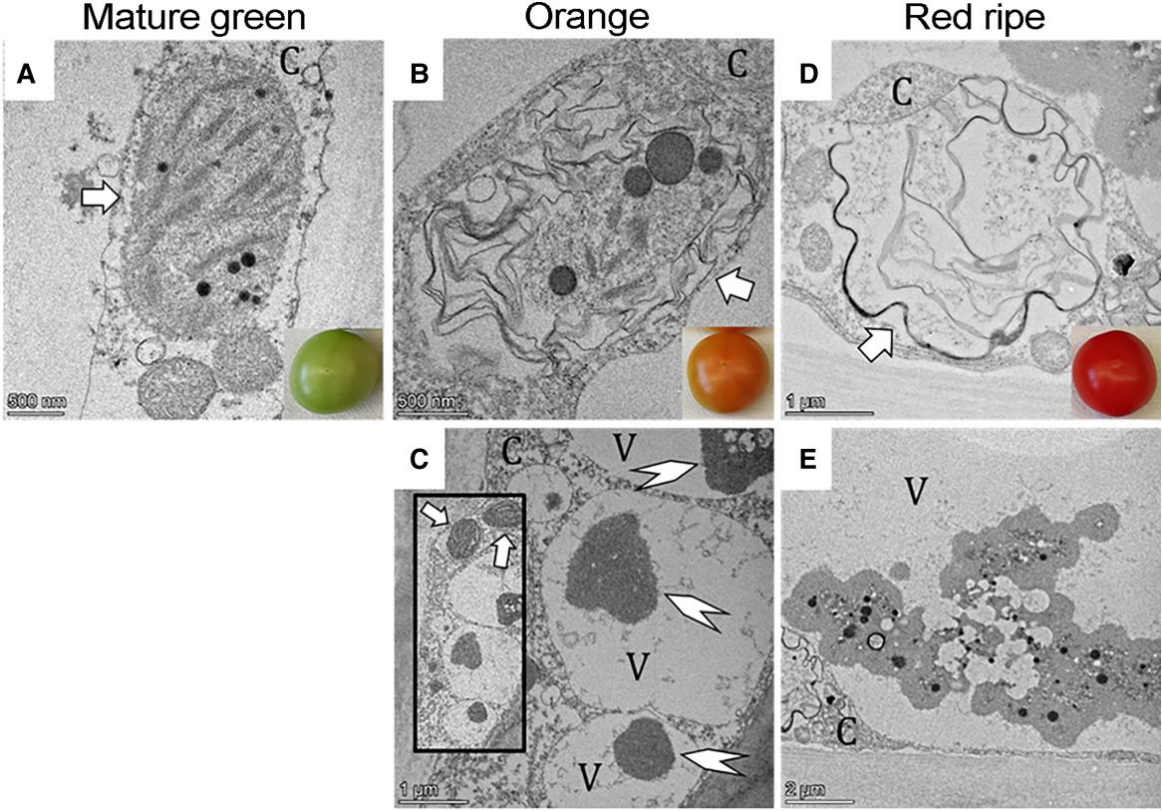
Fruit plastid evolution along ripening progression and their possible delivery to vacuoles. **A** to **E)** Transmission electron micrographs of ultrathin sections of tomato pericarp cells from three ripening stages as indicated. **A)** Chloroplast (arrow). **B)** Chromoplast (arrow). **C)** Vacuoles contain structures likely to be plastids (arrowheads) by judging the electron density of the cytoplasmic plastids in their vicinity (arrows in the inset). **D)** A chromoplast with signs of internal degradation (arrow). **E)** Vacuolar inclusions of what appear to be plastids remain (judging the internal plastoglobules). C, Cytoplasm. V, Vacuole lumen. Image credits: S. Mursalimov, A. Upcher, S. Michaeli.

In some fruits, such as tomatoes and bananas, color is used to assess the ripening stage and is determined by the ratio between two plastid types, chloroplasts and chromoplasts (Morelli et al. 2023). With ripening progression, chlorophyll-containing chloroplasts are gradually transformed into carotenoid-containing chromoplasts (Fig. 11). In Arabidopsis, SUPPRESSOR OF PPI1 LOCUS1 (SP1), a RING-type ubiquitin E3 ligase was found pivotal in the CHLORAD pathway (the section by Fang, Peixoto, and Jarvis herein). Recently, the tomato homologs SP1 and SP1-Like 2 (SPL2) were proposed as instrumental for chloroplast-to-chromoplast transition, suggesting that regulation of the plastid protein import machinery is vital for this plastid reformation (Ling et al. 2021).

#### Beyond the UPS: autophagy in ripening

Autophagy is another crucial cellular quality control and recycling mechanism involved in senescence and plant responses to stress. Therefore, it is a candidate target for the shelf-life extension of any of the fresh produce types. Autophagy is generally induced during cellular reprogramming (Rodriguez et al. 2020). Considering the dramatic cellular transformation during ripening (Fig. 11), it is surprising how little we know about autophagy`s role in this process. Recently, autophagy activity was shown to fluctuate during pepper and strawberry ripening (López-Vidal et al. 2020; Sánchez-Sevilla et al. 2021), and it was suggested to promote the ripening of the latter. Both these fruits are non-climacteric, meaning that their ripening is not associated with ethylene and respiration bursts (Perotti et al. 2023). On the other hand, in the climacteric tomato fruits, we have shown that autophagy restricts ripening by repressing ethylene production (Kumaran et al. 2023). It is tempting to speculate that the disparate function of autophagy in climacteric and non-climacteric fruits results from the differential role of ethylene between these two fruit types. It further highlights our insufficient knowledge of the interaction of autophagy with phytohormones (Liao et al. 2022). Transmission electron microscopy of tomato fruit pericarp cells suggests the vacuolar degradation of plastids during ripening (Fig. 11, C and E). Nonetheless, it still needs to be determined whether this is mediated via a macro- or microautophagy process (Izumi et al. 2017).

#### Proteolysis in postharvest regulation

Proteolysis pathways are highly selective toward specific targets within an explicit spatiotemporal environment (Clavel and Dagdas 2021), ideal for targeting individual traits. Can proteolysis pathways be harnessed for postharvest trait regulation and fresh produce shelf-life extension? To answer this, we first need to know whether there are applicable ways to induce or repress specific proteolysis pathways postharvest, avoiding fitness costs during the plant life cycle. For example, the constitutive knock-down of tomato *Autophagy-related 4* (*ATG4*) resulted in early leaf senescence and a considerably low fruit yield (Alseekh et al. 2022). However, when the same silencing was employed in a ripening-specific manner, these phenotypes were absent, and instead, the role of autophagy in ripening repression was revealed (Kumaran et al. 2023). This highlights the necessity of examining proteolysis pathways specifically within a postharvest context, and further, raises the challenge of uncovering and editing genetic segments that may be exclusively functional during postharvest. An alternative for genetic manipulation may be using compounds targeting proteolysis pathways that will be applied to fresh produce. Such compounds would need to be both human- and eco-friendly.

In conclusion, proteolysis pathways are pivotal for fresh produce's quality and shelf-life. Understanding the mechanisms governing these processes during postharvest may advance our ability to reduce food loss and waste and help ensure access to high-quality and nutritious produce.

## Roles of proteolysis in plant responses to biotic/abiotic signals

### How does ERAD function in model plants and crops?

**(Written by By Qian Chen, Qi Xie, and Feifei Yu)**

Abiotic and biotic stresses can trigger misfolded protein accumulation in the ER, causing ER stress. The unfolded protein response (UPR), ER-associated degradation (ERAD), and autophagy are three main mechanisms for relieving ER stress (Liu and Li 2014). Among them, ERAD involves the ER-located Ub modification system and the cytosolic proteasome degradation system for protein degradation (Romisch 2005). Traditionally, ERAD is responsible for identifying misfolded proteins in the ER lumen or membrane and facilitating their degradation (Romisch 2005). Recent studies have expanded the role of ERAD in the homeostasis of functional PM and cytoplasmic proteins to modulate various plant biological processes (Pan et al. 2020; Zhang et al. 2021; Li et al. 2023a, 2023b; Wang et al. 2023b). Here, we focus on progress in the study of ERAD in plant development and stress responses, broadening insights from model plants to crops.

**Figure 12.**
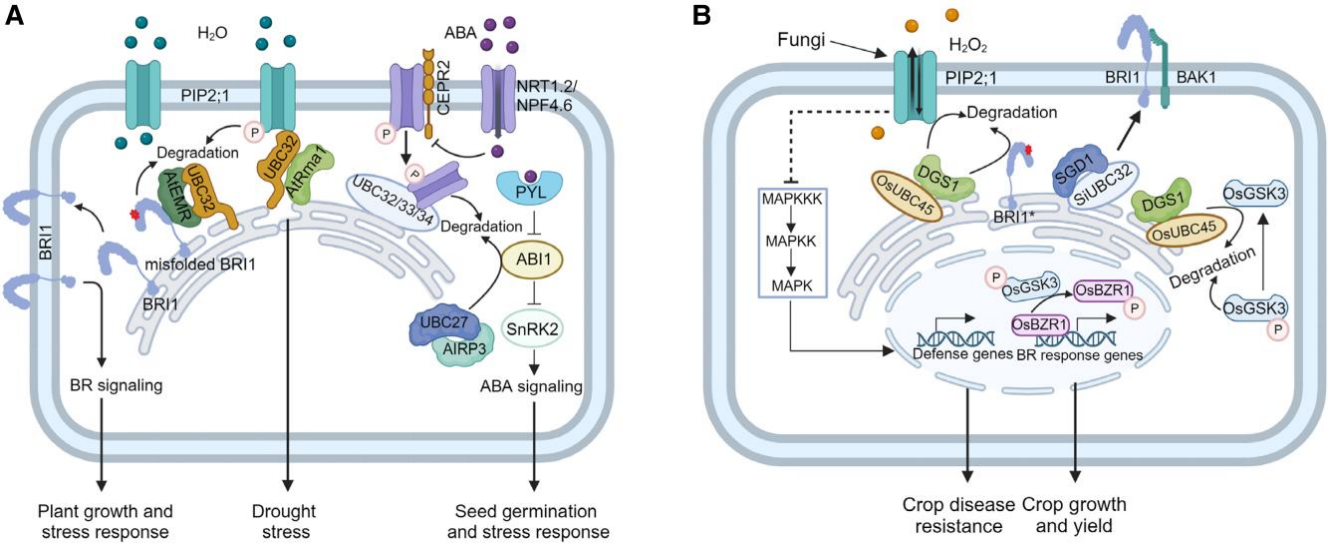
The role of ERAD in plant growth, crop yield, and stress response. **A)** The role of ERAD in stress response and phytohormone signaling in Arabidopsi*s*. The leucine-rich repeat receptor-like kinase (LRR-RLK) CEPR2 phosphorylates ABA importer NRT1.2/NPF4.6, inhibiting its ability to import ABA. The phosphorylated NRT1.2/NPF4.6 is then transported to the ER for ubiquitylation and degradation, mediated by UBC32 and its homologs UBC33 and UBC34. ABA inhibits the CEPR2-mediated phosphorylation of NRT1.2/NPF4.6. ABA receptor PYL recognizes ABA and initiates the transduction of ABA signaling. UBC27 and AIRP3 act as an E2–E3 pair to activate ABA signaling and enhance drought tolerance by promoting the ubiquitylation and degradation of ABI1. Moreover, UBC32 collaborates with AtEMR1 to facilitate the degradation of misfolded BRI1, thereby influencing BR signaling under ER stress conditions. During drought stress, Rma1 and UBC32 work together to enhance drought tolerance by promoting the degradation of phosphorylated aquaporin PIP2; 1. **B)** The role of ERAD in crop yield and disease resistance. The ERAD-related E2-E3 pair, OsUBC45/SiUBC32-DGS1/SGD1 in rice and millet, enhances yield by regulating BR signaling via distinct mechanisms. They enhance BR signaling by reducing the protein level of misfolded BR receptor BRI1 (in rice) or increasing the protein level of folded BRI1 (in millet). Additionally, the E2–E3 pair also promotes the Ub-dependent degradation of OsGSK3, a negative regulator of BR signaling. Under fungi attack, OsPIP2; 1 facilitates the translocation of H_2_O_2_ from the cytoplasm to the apoplast, negatively regulating pattern-triggered immunity (PTI). OsUBC45 and DGS1 promote the degradation of OsPIP2; 1, enhancing rice resistance to disease.

The role of ERAD in plant abiotic stress response has primarily been studied in Arabidopsis (Fig. 12A). A key ERAD component, UBC32, an ER membrane-anchored Ub-conjugating enzyme (E2), is transcriptionally induced by salt and drought stresses (Cui et al. 2012). UBC32 plays a role in the brassinosteroid (BR)-mediated salt stress response. Park et al. (2018) showed that the soluble ERAD component, ERAD-mediating RING finger protein (AtEMR), forms a complex with UBC32, negatively regulating salt stress resistance. Additionally, UBC32 cooperates with the RING-type E3 ligase Rma1 as an E2–E3 pair, enhancing plant drought tolerance by facilitating the degradation of aquaporin PIP2; 1 (Chen et al. 2021b). UBC32, together with its homologs UBC33 and UBC34, also participates in ABA signaling by degrading the phosphorylated ABA transporter NITRATE TRANSPORTER 1.2/PEPTIDE TRANSPORTER 4.6 (NRT1.2/NPF4.6) (Zhang et al. 2021). Since ABA is crucial in plant drought response, it is still an open question whether UBC32 responds to drought via ABA signaling. Another E2, UBC27, an ortholog of the yeast ERAD component Ubc1p, interacts with ABA-INSENSITIVE RING PROTEIN 3 (AIRP3). The UBC27-AIRP3 interaction is enhanced by ABA which leads to ubiquitylation and degradation of ABA co-receptor ABA-INSENSITIVE 1 (ABI1), thus activating ABA signaling and improving drought tolerance (Pan et al. 2020). These findings from Arabidopsis indicate that ERAD plays crucial roles in stress-related hormone signaling and plant adaptation to environmental stress.

Although significant progress has been made in understanding ERAD in model plants, our understanding of ERAD in crop stress response and growth is limited. In *Medicago falcata*, plant-speciﬁc E3 ligase MfSTMIR participates in the ERAD pathway via interacting with MtUBC32 to relieve ER stress under salt stress (Zhang et al. 2019b). This finding underscores the significance of ERAD pathways in crop salt stress response. However, further studies are required to explore their roles in other stress conditions.

Recent studies have highlighted the role of ERAD in biotic stress resistance in a few crops (Fig. 12B). In rice, overexpression of *OsUBC45*, the ortholog of Arabidopsis UBC32, exhibited improved resistance to rice blast and bacterial leaf blight by promoting the degradation of OsPIP2; 1, which attenuates disease resistance by mediating the translocation of H_2_O_2_ from the cytosol to the apoplast (Wang et al. 2023b). The ERAD-related RING-type E3 Ub ligase Decreased Grain Size 1 (DGS1) improved resistance to rice blast, by forming an E2–E3 pair with OsUBC45 to enhance the degradation of OsPIP2; 1 (Wang et al. 2024). In foxtail millet (*Setaria italica*), the overexpression of the ERAD-related RING-type E3 Ub ligase Small Grain and Dwarf (SGD1) also increased blast resistance, though the mechanism remains undefined (Tang et al. 2023). Whether SiUBC32 in millet also contributes to disease resistance needs to be further explored.

Significant advances have been made in understanding the crucial role of ERAD-related E2–E3 in grain yield in graminaceous cereals (Fig. 12B). In rice, SMALL GRAIN 3 (SMG3), the other name of OsUBC45, works together with E3 ligase DGS1 to positively regulate grain size by facilitating the degradation of the misfolded BR receptor BRI1 (Li et al. 2023a). Intriguingly, the same E2–E3 pair OsUBC45/SMG3-DGS1 also enhances rice yield by targeting GLYCOGEN SYNTHASE KINASE 3 (OsGSK3), a negative component in BR signaling, for ubiquitylation-dependent degradation (Gao et al. 2019b; Wang et al. 2023b). In millet, the SGD1–SiUBC32 pair also boosts yield by strengthening BR signaling. They catalyze Ub attachment to BRI1 but lead to an accumulation of functional BRI1 rather than its degradation (Tang et al. 2023). Additionally, the role of SGD1 in regulating seed size is also conserved in wheat (*Triticum aestivum*) and maize (Tang et al. 2023). These studies in crops demonstrate that this specific E2–E3 pair could contribute to crop yield by enhancing BR signaling through regulating both positive and negative components involved in BR recognition and signal transduction. Further efforts should explore the role of DGS1 orthologs in wheat and maize disease resistance and determine whether the SMG3/OsUBC45/SiUBC32-DGS1/SGD1 pair acts as an E2–E3 pair, targeting different substrates to improve both yield and disease resistance in staple crops. Moreover, *OsUBC11* in rice, the ortholog of *AtUBC7* in Arabidopsis, which encodes ERAD components, is implicated in root development at the seedling stage by affecting auxin signaling (Han et al. 2023), implying the potential roles of other ERAD components in crop development.

ERAD components play a significant role in phytohormone signaling, including ABA, BR, and auxin pathways, and crosstalk among these hormones has been known to balance plant development and stress response (Wang et al. 2020; Zhang et al. 2021; Song et al. 2023; Tang et al. 2023; Li et al. 2023a, 2023b; Wang et al. 2023b; Yu and Xie 2024). It is important to note that although the function of ERAD is largely conserved across eukaryotes, there may be differences between model plants and crops. For instance, the mutants of *UBC32* in Arabidopsis show minimal impact on plant growth and seed size (Cui et al. 2012), whereas its orthologs in rice and millet are essential factors for both growth and yield (Li et al. 2023a; Tang et al. 2023; Wang et al. 2023b), which may be partially explained by the distinct BR signaling between Arabidopsis and rice. Therefore, it is crucial to reveal the specific role of other ERAD components, such as the E3 Ub ligase HRD1 and DOA10 in crop growth and environmental stress interaction, which will provide us the possibility of utilizing them in crop breeding.

### How is chloroplast-associated protein degradation (CHLORAD) regulated in response to developmental and environmental cues?

**(Written by Jun Fang, Bruno Peixoto, and R. Paul Jarvis)**

Chloroplasts are essential plant organelles, not only for photosynthesis but also for the biosynthesis of many important primary and secondary metabolites (Lopez-Juez and Pyke 2005; Sun and Jarvis 2023). Chloroplasts originated through endosymbiosis from an ancient cyanobacteria-like photosynthetic prokaryote (Reyes-Prieto et al. 2007), and the modern organelles retain a functional genome with roughly 100 protein-coding genes. However, most endosymbiont genes were transferred to the host nuclear genome during evolution. Consequently, >90% of the ∼3,000 different chloroplast proteins are nucleus-encoded and must be imported following synthesis as precursors in the cytosol (Li and Chiu 2010; Shi and Theg 2013; Sun and Jarvis 2023).

**Figure 13.**
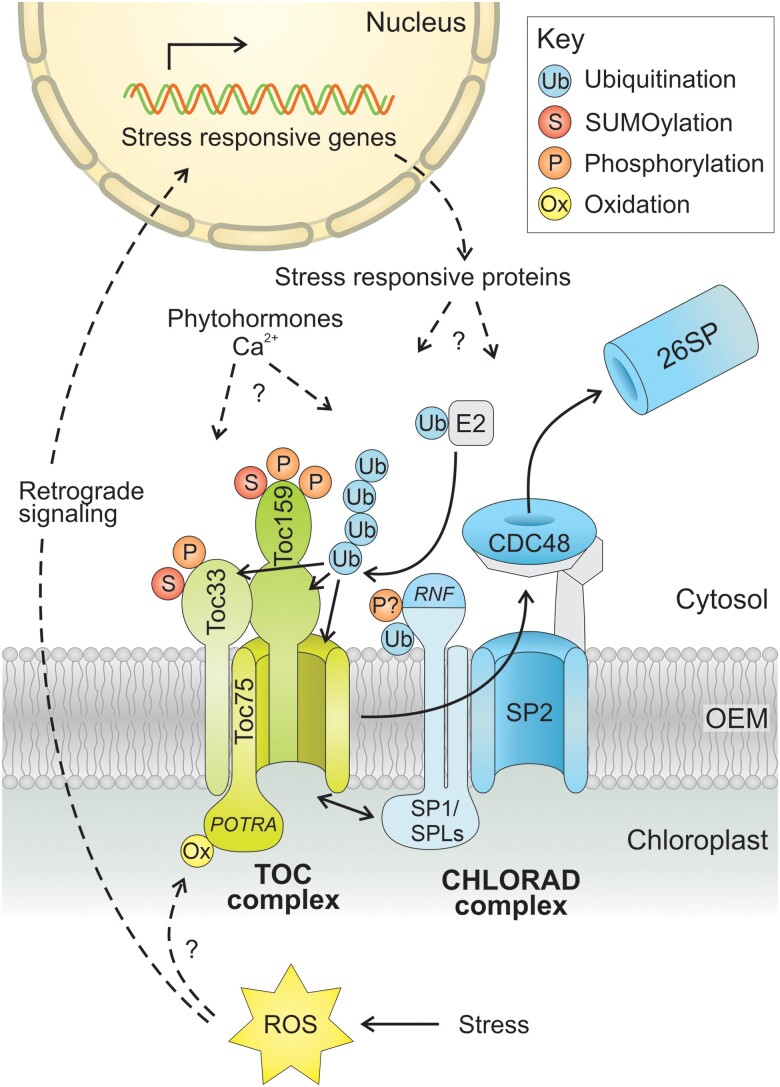
Possible mechanisms of regulation of chloroplast-associated protein degradation. CHLORAD is a UPS pathway that selectively degrades chloroplast-resident proteins, including the TOC apparatus that is responsible for protein import. The SP1 Ub E3 ligase recruits E2 Ub-conjugating enzyme [via its RING finger (RNF) domain] to direct the ubiquitylation of TOC proteins, which are then degraded through the combined action of the SP2 retrotranslocation channel, the CDC48 ATPase motor, and the cytosolic 26S proteasome (26SP); in this, the activity of SP1 is modulated by the action of SP1-like components (SPLs). Thus, CHLORAD exerts important control over protein import and the organelle's proteome and functions. Such control is responsive to developmental and environmental cues through unclear mechanisms. Under different conditions, phytohormone, Ca^2+^, or reactive oxygen species (ROS) signaling might regulate the activity of CHLORAD, and this is possibly mediated through post-translational modification of the CHLORAD machinery or its TOC apparatus targets, and/or through retrograde signaling and stress-responsive proteins. Post-translational modifications that are potentially involved in such regulation are indicated.

Import of these precursors into chloroplasts requires translocons in the outer and inner chloroplast envelope membranes (TOC and TIC, respectively). The TOC complex exists in different, client-specific subtypes (Li and Chiu 2010; Shi and Theg 2013). The main TOC subtype, comprising the receptors Toc33 and Toc159 and the channel Toc75, preferentially imports precursors of the photosynthetic apparatus. In contrast, a minor TOC subtype with a different complement of receptors (Toc34, Toc132, and Toc120) tends to import non-photosynthetic, housekeeping precursors (Kessler and Schnell 2009; Li and Chiu 2010). The TOC machinery acts as the gateway controlling entry of the desired types of precursors. The guardian that regulates the TOC machinery is the so-called chloroplast-associated protein degradation (CHLORAD) system. CHLORAD is an arm of the UPS, and it targets TOC proteins for ubiquitylation and degradation, thereby controlling the import of precursor proteins. A separate system proteolytically removes unimported precursors to prevent their cytosolic accumulation (Lee et al. 2009, 2016; Grimmer et al. 2020), although whether or how this system is coordinated with CHLORAD is not known. The CHLORAD system is composed of the RING-type E3 Ub ligase SUPPRESSOR OF PPI1 LOCUS1 (SP1), an Omp85-type β-barrel protein SP2, and a cytosolic AAA-ATPase motor CDC48 (Ling et al. 2012; Ling et al. 2019). As we summarize below, CHLORAD plays important roles both developmentally and under stress conditions. However, there are major outstanding questions concerning how CHLORAD is regulated by different developmental and environmental cues (Fig. 13).

Chloroplasts are the best-known type of plastid, but there are several other plastid types in non-green plant tissues. A remarkable feature of these different plastid types is their ability to interconvert in response to developmental or environmental signals. Such plastid-type interconversions involve the remodeling of the plastid proteome, which is controlled at least in part by differential regulation of protein import, particularly at the TOC machinery (Jarvis and López-Juez 2013; Nellaepalli et al. 2023). CHLORAD degrades TOC proteins to facilitate their replacement by others, thereby controlling plastid protein import, the organellar proteome, and plastid transitions.

Work in Arabidopsis showed that loss of SP1 leads to delayed de-etiolation and leaf senescence, whereas overexpression of SP1 promotes these processes by accelerating plastid transitions (i.e. etioplast-to-chloroplast and chloroplast-to-gerontoplast transitions, respectively). Such functions of CHLORAD appear well-conserved among plant species, as its manipulation similarly affects leaf senescence in tomato, as well as fruit ripening during which chromoplast formation occurs (Ling et al. 2012; Ling et al. 2021). Recently, two homologs of SP1, namely SP1-like1 (SPL1) and SPL2, were shown to regulate CHLORAD in an antagonistic manner (Mohd Ali et al. 2023). While SPL2 exhibits partial redundancy with SP1, SPL1 negatively regulates SP1 potentially through competitive interaction with other factors (Ling et al. 2021; Mohd Ali et al. 2023). Both SPL proteins are important for leaf senescence, like SP1. However, it remains unclear how CHLORAD perceives developmental signals, and how it selectively degrades different TOC components during different developmental phases.

Different TOC subtypes are regulated transcriptionally in different plant tissues and developmental stages (Demarsy et al. 2014). In contrast, the *SP1* and *SP2* genes show comparable expression profiles across different tissues and stages (Ling et al. 2019). Therefore, it is likely that SP1 and SP2 are regulated post-translationally. Studies have shown that TOC receptors can be phosphorylated in vitro and in vivo, and such phosphorylation may inhibit TOC complex assembly, GTP binding, and precursor binding (Demarsy et al. 2014). For example, physiological analyses suggested that phosphorylation at residue S181 reduces Toc33 activity and impairs chloroplast biogenesis at early developmental stages, but not later growth (Aronsson et al. 2006; Oreb et al. 2007). A kinase at the outer chloroplast membrane (KOC1) phosphorylates the A-domain of Toc159 in vitro and contributes to efficient protein import and chloroplast biogenesis during de-etiolation (Zufferey et al. 2017). Differential phosphorylation of Toc159 has also been described when the carbon-sensing kinase SnRK1 (sucrose nonfermenting 1-related protein kinase 1) is genetically manipulated, with SnRK1α1 gain-/loss-of-function lines showing higher/lower levels of phosphorylated Toc159, respectively (Nukarinen et al. 2016; Cho et al. 2016a). Moreover, phosphorylation of Toc159 family proteins by SnRK2, and reduced import efficiency in an ABA biogenesis deficient mutant, implies crosstalk between ABA signaling and protein import regulation (Zhong et al. 2010; Wang et al. 2013a). There is presently no information on whether CHLORAD components undergo differential phosphorylation at different developmental stages, although SP1 and SP2 are predicted to have 18 and 15 phosphorylation sites, respectively (Chen et al. 2023a). Phosphorylation may regulate E3 ligase activity, substrate recognition, or substrate/ligase interaction (Hunter 2007).

Besides its developmental role, CHLORAD is also critically important for abiotic stress tolerance in plants (Ling and Jarvis 2015). Under stress conditions, chloroplasts overproduce reactive oxygen species (ROS), harmful photosynthetic byproducts that can oxidize macromolecules and affect organellar structural and functional integrity (Li and Kim 2022). During stress, CHLORAD degrades TOC proteins to limit the import of photosynthesis-related proteins, thereby suppressing photosynthetic activity and reducing ROS production and photo-oxidative damage (Ling and Jarvis 2015). In addition to their toxicity, ROS also function as signaling molecules via the redox modification of specific amino acid residues, for example, at cysteine thiol groups (Li and Kim 2022). Evidence suggests that conserved cysteines in TOC components may be regulated by redox modification, thereby influencing protein import (Kessler and Schnell 2009; Balsera et al. 2010). Interestingly, Toc75 was found to be oxidized at C219 within its polypeptide transport-associated (POTRA) domain, after hydrogen peroxide treatment (Doron et al. 2021). While SP1, SPL1, and SPL2 share several conserved cysteines, it is currently unknown whether these cysteine residues are essential for the function or involved in redox-mediated regulation (Ling et al. 2012; Ling et al. 2021). It will be interesting to investigate whether the CHLORAD apparatus is regulated by redox modification directly or by altered affinity toward redox-modified/unmodified TOC proteins. This might enable rapid limitation of the import of photosynthesis proteins and further ROS production.

Aside from such direct effects, ROS also induce stress-responsive gene expression changes by transmitting signals from chloroplasts to the nucleus. This is referred to as retrograde signaling, and it plays important roles in maintaining cellular homeostasis and acclimation to stressful environments (Li and Kim 2022). For example, singlet oxygen (^1^O_2_) oxidizes β-carotene to produce β-cyclocitral, which induces detoxification-related nuclear genes via the SCARECROW-LIKE 14 (SCL14) transcription factor, or salicylic acid (SA)-responsive genes through a chloroplast SA-synthesis enzyme, isochorismate synthase1 [ICS1 (Lv et al. 2015; D’Alessandro et al. 2018)]. Chloroplast-resident EXECUTER1 (EX1) protein is also oxidized by ^1^O_2_ at its W643 residue, inducing ^1^O_2_-responsive gene expression (Dogra et al. 2019). Thus, the question arises: Does ROS-induced retrograde signaling play a role in regulating CHLORAD? One possibility is that the expression of *SP1* and *SP2* is regulated under abiotic stresses. However, differential expression of these genes was not observed under several abiotic stresses (Hruz et al. 2008; Coolen et al. 2016; Garcia-Molina and Pastor 2023). Another possibility is that CHLORAD is a target of stress-responsive proteins regulated by retrograde signaling, for example, proteins involved in stress-related phytohormone signaling or ROS-triggered responses (Li and Kim 2022).

In addition to ROS, cytosolic calcium, various phytohormones including ABA, and diverse kinase subfamilies such as type 2C protein phosphatase (PP2C), SnRK2, SnRK1, and mitogen-activated protein kinases (MAPKs) also play major signal transduction roles during abiotic stress (Belda-Palazón et al. 2020; Zhang et al. 2022). Therefore, it is conceivable that CHLORAD perceives stress signals transduced from the PM or cytosol as part of an integrated cellular stress response. The CALCINEURIN B-LIKE 10 (CBL10) protein, a member of the CBL family that perceives and transmits Ca^2+^ signals to CBL-interacting protein kinases, was found to interact with Toc34 and negatively regulate its GTPase activity (Cho et al. 2016b). As mentioned above, ABA signaling can influence the phosphorylation of Toc159 family proteins. Thus, emerging evidence suggests possible crosstalk between Ca^2+^ and hormone signaling and protein import regulation, although whether CHLORAD plays any role in this is unknown. It is clear that post-translational modification of substrate proteins, such as acetylation, phosphorylation, and SUMOylation, can alter substrate recognition by RING E3 ligases (Metzger et al. 2014). In this regard, it is noteworthy that the E2 SUMO conjugase SCE1 is implicated in the SUMOylation of TOC proteins to promote their degradation, possibly through CHLORAD activity (Watson et al. 2021). However, it is unclear whether SUMOylation affects substrate recognition by SP1 or some other step such as CDC48 recruitment.

Many important questions remain concerning CHLORAD action and the regulation of chloroplast protein import. We look forward to seeing significant new light shed in this intriguing area in the future. Because of its importance for plastid development and plant stress responses, a greater understanding of CHLORAD regulation may prove invaluable in efforts to improve crop performance concerning yield, quality, and stress resilience.

### How does autophagy contribute to drought tolerance?

**(Written by Diane C. Bassham)**

Autophagy, a pathway leading to the degradation of cellular components in the vacuole, is activated by numerous abiotic stresses, including drought (Agbemafle et al. 2023). The activities of two major kinases are responsible for regulating autophagy under many conditions: the Target of Rapamycin (TOR) complex, a negative regulator, and SnRK1, a positive regulator. Downstream of these kinases, a suite of ATG (autophagy-related) proteins function in the de novo production of double-membrane vesicles termed autophagosomes that enwrap the cargo to be degraded. The cargo is delivered into the vacuole, degraded by vacuolar hydrolases, and the degradation products are recycled into the cytoplasm (Agbemafle et al. 2023). Disruption of autophagy by mutating core autophagy genes or by using inhibitors decreases drought tolerance (Liu et al. 2009). Overexpression in Arabidopsis of *ATG* genes derived from crop species leads to improved drought tolerance (Li et al. 2019, 2015; Fu et al. 2020; Chen et al. 2022; Yue et al. 2022), as does overexpression of *ATG18* and *ATG8* homologs in apple (Sun et al. 2018; Jia et al. 2021). These data indicate a critical function for autophagy in survival during drought, including in economically important crop species, but the mechanisms by which autophagy acts to allow survival remain an open question.

**Figure 14.**
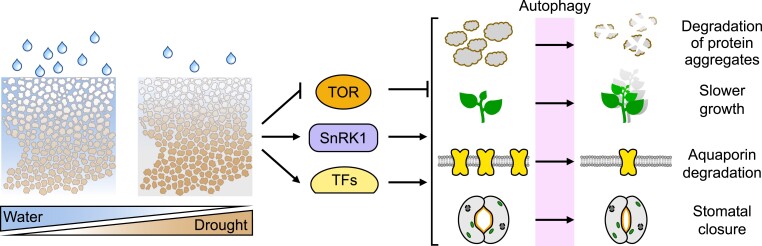
Possible mechanisms by which autophagy regulates drought tolerance. Autophagy is activated by drought stress via the TOR complex, SnRK1, and transcriptional pathways. Activation of autophagy may lead to increased degradation of protein aggregates and aquaporins, and decreased growth and stomatal aperture, in turn aiding tolerance of drought conditions.

Several pathways by which plants activate autophagy during the drought have been identified, although how they work together remains unclear (Fig. 14). In a number of plant species, *ATG* genes are upregulated by drought, and autophagy activity increases (Tang and Bassham 2022; Agbemafle et al. 2023); several transcription factors have been identified that can control the expression of these *ATG* genes during drought (Agbemafle et al. 2023). As for other stresses, TOR and SnRK1 kinases are critical in activating autophagy during drought, with repression of TOR activity and increased SnRK1 activity leading to activation (Pu et al. 2017; Soto-Burgos and Bassham 2017; Chen et al. 2017a). It is becoming clear that sulfide signaling also regulates stress responses such as autophagy (Jurado-Flores et al. 2023). The core autophagy protein ATG4 is persulfidated and inactivated upon osmotic stress or ABA treatment, leading to a downregulation of autophagy (Laureano-Marín et al. 2020), potentially preventing over-activation and cell death.

While it is well established that autophagy is activated during drought and aids in drought tolerance, major questions remain about the pathways by which autophagy contributes to tolerance. Several distinct mechanisms have been proposed, but which ones predominate and how these mechanisms are integrated is not yet known, and are crucial topics for future research.

#### Degradation of oxidized and/or aggregated proteins

Several reports indicate that autophagy is important for clearing aggregated or oxidized proteins during drought and other stresses (Zhou et al. 2013b; Sun et al. 2018) and that the activities of antioxidant pathways correlate with autophagy activity (Li et al. 2019; Jia et al. 2021). This potentially could relieve cytotoxic stress caused by the accumulation of damaged proteins and other macromolecules.

#### Regulation of aquaporin activity

Aquaporins are channels that control the flux of water and other small molecules across membranes. In both Medicago (Li et al. 2020a) and Arabidopsis (Hachez et al. 2014), PM aquaporins are recognized by selective autophagy receptors and degraded during drought, although the receptors in each species are distinct. This degradation is proposed to reduce water loss from cells and improve drought tolerance.

#### Regulation of stomatal dynamics

Interesting recent findings implicate autophagy in stomatal dynamics, which are critical to prevent loss of water in conditions of water deficit. Reactive oxygen species signal in response to environmental stress to inhibit stomatal opening and/or promote closing; ROS homeostasis in guard cells therefore may be important for drought tolerance. Autophagy is required for maintaining basal levels of ROS, and Arabidopsis mutants defective in autophagy have high ROS levels in guard cells, with defects in stomatal movement (Yamauchi et al. 2019). Interestingly, ABA responses, guard cell opening, and ROS homeostasis have all been linked to regulation by protein persulfidation, suggesting that the integration of drought responses may involve sulfide signaling (Jurado-Flores et al. 2023).

#### Regulation of growth

The transcription factor BES1 controls growth in response to brassinosteroid signaling. During drought, BES1 is degraded by selective autophagy via the DSK2 receptor, leading to decreased growth and increased drought tolerance (Nolan et al. 2017). In general, water use efficiency (i.e. the ratio between water used by the plant and that lost by transpiration) is correlated with leaf area, but unexpectedly not with stomatal density or ABA levels, suggesting a complex relationship between plant size, water use, and drought tolerance (de Ollas et al. 2023). This is demonstrated in the case of Arabidopsis *cost1* (constitutively stressed) mutants, which have greatly reduced growth, constitutive autophagy, and are highly drought tolerant (Bao et al. 2020). The drought tolerance requires active autophagy, and COST1 inhibits autophagy by interacting with the autophagy machinery. During drought, COST1 is degraded, releasing the inhibition of autophagy and increasing drought tolerance. Intriguingly, recent work showed that *cost1* mutants are drought tolerant because they use less water due to their extreme dwarfism and that when grown together in the same pot, no drought tolerance is observed (Ginzburg et al. 2022). However, the *cost1* drought tolerance phenotype can be rescued by blocking autophagy, but the growth phenotype cannot, indicating that plant size and drought tolerance can be uncoupled in this mutant (Bao et al. 2020).

These data all indicate an important role for autophagy in drought responses, but also raise many questions about the precise role of autophagy in these responses, the mechanisms by which drought is perceived and autophagy is activated, and the integration of stress, growth, and developmental pathways to allow plant survival. New approaches including the physiological characterization of higher-order mutants in different aspects of the drought response, identification of additional factors, for example by protein–protein interaction, that link autophagy to drought tolerance, and non-targeted approaches such as suppressor screens are needed to determine the relationships between identified pathways and to clarify direct and indirect contributions of proteins and pathways to drought tolerance. The activity and role of autophagy in the phenotypes of known drought-tolerant or sensitive mutants also deserve investigation.

### How does the fine-tuning of proteasome regulation impact the trade-off between growth and defense?

**(Written by Suayib Üstün)**

Trade-offs, situations when a beneficial change in one feature comes with a detrimental change in another, are inherent to life (Garland 2014). One of the most prevalent examples is the growth-defense trade-off in plant–microbe interactions. Under changing environmental conditions, and when resources are scarce, plants must decide between growth or defense. Growth-defense trade-offs are triggered by changes in the nutrient status and by the activation of pathways with contrasting functions, e.g. either promoting or limiting growth. Thus, the trade-off between growth and defense has an enormous impact on plant survival, reproduction, plant fitness, and crop yields (Huot et al. 2014). As such, it is not surprising that plant hormones, transcription factors, and kinases that sense the nutrient status of a cell, all play roles in balancing growth-defense trade-offs (Lozano-Durán et al. 2013; Huot et al. 2014; De Vleesschauwer et al. 2018; Margalha et al. 2019). Proteostasis, the balance between protein biosynthesis and degradation, has a huge impact on the growth-defense trade-off. Considering that approximately 80% of protein degradation in plants is mediated by the UPS, and given the role of the UPS in plant-microbe interactions, cell survival, and growth (Raffeiner et al. 2023; Langin et al. 2023a), it is evident that the UPS plays a major role in the growth-defense trade-off. How does the proteasome directly and indirectly influence the trade-off between growth and defense?

#### The role of the UPS in cellular homeostasis

The UPS not only recycles proteins as a housekeeper but also has essential roles in controlling developmental processes and stress responses by fine-tuning the amount of central regulatory proteins (Raffeiner et al. 2023). Proteasome mutants often display pleiotropic phenotypes, perhaps related to the role of the UPS in central processes in plant growth and development, such as balancing cell division and expansion in plants (and see the section above by Bednarek), and plant response to environmental conditions, including biotic and abiotic stresses (Kurepa et al. 2009; Langin et al. 2023a, and see above section by Bassham).

Mounting evidence suggests that the UPS is involved in degrading organelle-associated proteins to alleviate stress conditions (Clavel and Dagdas 2021, and see above sections by Murcha and van Wijk). As chloroplasts are the most essential energy source in plants, impairment of proteasome-mediated chloroplast quality control can be expected to have a dramatic effect on plant fitness. Indeed, severe proteasome stress induced by high concentrations of the proteasome inhibitor MG132 inhibits root growth (Sheng et al. 2012). However, mild proteasome stress was shown to enhance root growth (Sheng et al. 2012) and can also have a positive effect on photosynthesis and plant performance (Grimmer et al. 2020), suggesting that we are missing some puzzle pieces in our understanding of the role of the UPS in the control of plant growth.

#### Why is the proteasome manipulated during plant-microbe interactions?

The proteasome might be considered an ideal target for manipulation by microbes to impact as many pathways and compartments as possible (Langin et al. 2020, 2023a). The proteasome controls plant immune reactions from pathogen perception to execution and thus is a master regulator of plant immunity (Adams and Spoel 2018; Langin et al. 2023a). It is known that loss of various proteasome subunits leads to increased susceptibility toward pathogens (Üstün et al. 2016; Langin et al. 2023a). The inactivation of the proteasome seems to be in general beneficial for most pathogens, although it leads to growth penalties and developmental alterations. Thus, various pathogens, from bacteria to viruses, directly target and inactivate the proteasome to subvert many cellular processes (Langin et al. 2020).

However, there are also contrasting effects on the proteasome during plant-microbe interactions. Although certain pathogens suppress the function of the proteasome to cause disease, the same pathogens also activate the proteasome to degrade central regulators of plant immunity (Langin et al. 2020). How these contrasting functions work together to influence the proteasome remains to be understood but inactivation and activation likely occur in parallel during pathogen attack. Nevertheless, in a simplified scenario, proteasome activation might be the way to combat disease. Indeed, during various pathogen infections or SA treatment, transcription as well as translation of proteasome subunits are induced, which can be explained in two ways: (i) Given the direct role of some subunits in plant defense reactions (Hatsugai et al. 2009; Üstün et al. 2016), proteasome subunits are transcriptionally and translationally induced as a form of defense reaction or (ii) pathogens directly induce the expression of the proteasome to hijack the proteasome. If we think about the scenario, (i) strengthening the proteasome should lead to resistance without affecting growth. Intriguingly, a recent study discovered a natural allele of proteasome maturation factor UMP1, displaying enhanced proteasome abundance and activity, leading to resistance to multiple pathogens in rice (Hu et al. 2023). While pathogen infection is restricted, defense reactions are increased without any yield penalty. Taken together, activation of the proteasome circumvents the growth-defense trade-off. Uncoupling growth and defense trade-offs have been shown in very rare cases, e.g. regulated expression of SA master regulator NPR1 using uORF-mediated translational control (Xu et al. 2017). It appears that utilizing proteostasis seems to be the key to engineering plant disease resistance without fitness costs.

#### Does proteasome activation balance growth-defense trade-offs?

**Figure 15.**
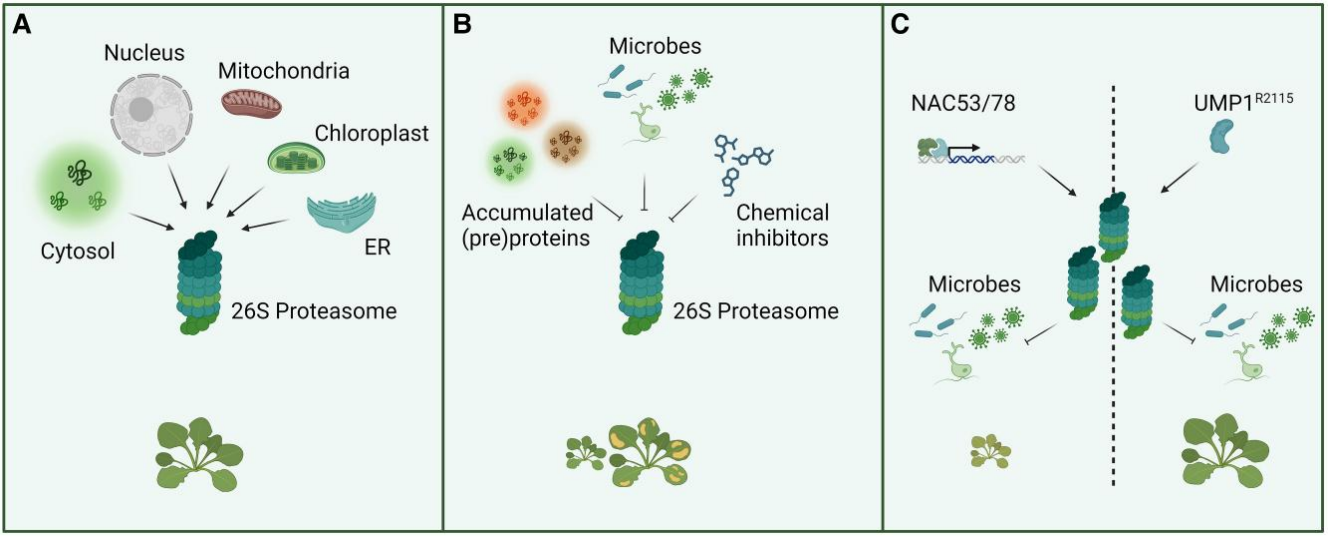
The proteasome influences the growth-defense trade-off. **A)** The proteasome degrades substrates from various cellular compartments and organelles to maintain cell survival and optimal growth. **B)** Proteins that accumulate due to stress conditions or accumulating preproteins from organelles or microbes as well as chemical inhibitors can interfere with proteasome function leading to proteotoxic stress. This will cause growth penalties, impact survival, and in the case of microbes cause disease. **C)** On the one hand, a natural allele of proteasome maturation factor UMP1R2115 results in more proteasome abundance and activity improving resistance to multiple pathogens without growth penalties. On the other hand, proteasome activation can be achieved by the transcription factor pair NAC53 and NAC78. Whether this transcriptional activation of the proteasome results in resistance to pathogens and how it impacts plant growth remains to be discovered. The figure was created using BioRender (https://biorender.com).

But would proteasome activation always bypass the growth-defense trade-off? Activation of the proteasome is governed by a chaperone network, including UMP1, that is essential for proteasome assembly and function. Before this, proteasome subunit genes need to be expressed (Marshall and Vierstra 2019). In Arabidopsis, two NAC transcription factors, NAC53 and 78, act in concert to activate the gene expression of proteasome subunits (Gladman et al. 2016). Considering the broad role of NACs in many cellular pathways, it is likely that both transcription factors might have other targets beyond the proteasome. Indeed, both transcription factors have been found to additionally target and repress photosynthesis-associated nuclear genes during proteotoxicity affecting the energy status of the cell (Langin et al. 2023b). The trade-off between proteasome activation and photosynthesis downregulation seems to be a general feature as it occurs in response to various environmental and developmental cues (Langin et al. 2023b). In this scenario, transcriptional upregulation of proteasome subunits might be considered a defense strategy to restrict pathogens by repressing photosynthesis when pathogens suppress the proteasome (Fig. 15).

Although the role of the proteasome in growth and defense has been extensively studied, many questions remain elusive: (1) Does the magnitude of proteasome activation and de-activation decide how the growth-defense trade-off is influenced? (2) Does the proteasome act as a trap in plant-microbe interactions leading to growth penalties to limit pathogens? (3) How can we explain contrasting effects on the proteasome during pathogen infection? (4) Can we engineer plants that evade the growth-defense trade-off using the transcriptional activation of the proteasome by NAC53/78? Addressing these questions in the future will reveal how up- and downregulation of the proteasome during plant–microbe interactions integrates different signals to balance the trade-off between growth and defense.

### Why are there so many peptidases in plants, particularly in the subtilase family?

**(Written by Annick Stintzi and Andreas Schaller)**

Plants devote a large fraction of their proteome to proteolysis. Adding to the proteasome and the multi-component UPS, there are several hundred peptidases in plants, for example, 685 peptidases in Arabidopsis (MEROPS database https://www.ebi.ac.uk/merops/). Here, we discuss whether the expansion of peptidase families is driven by functional diversification or, more specifically, by the specialization of peptidases for certain substrate proteins, for specific processing sites, or by distinct mechanisms of regulation. We pose these questions for subtilases (SBTs), the S8 family of serine peptidases and one of the largest and most studied peptidase families in plants (Schaller et al. 2018).

#### Specialization for substrate proteins

Plant-specific SBTs (five of the seven SBT clades in tracheophytes) originated from a single event of horizontal gene transfer from a bacterial donor to streptophyte algae (Xu et al. 2019). Early gene duplication resulted in two copies, one evolving into the *SBT2* clade, the other one ancestral to clade1 and clades 3 to 5. While the *SBT2* lineage remained well-conserved with low copy numbers throughout land plant evolution, the *SBT1* and *SBT3-5* lineages underwent massive expansion (Xu et al. 2019). Interestingly, the size of individual clades differs dramatically between angiosperm taxa, suggesting that some of the gene duplication events occurred comparatively recently.

The *SBT1* clade, for example, is rather small in Arabidopsis with only 9 members, compared to 61 in tomato (*Solanum lycopersicum*) and an average of 21.7 across land plants (Taylor and Qiu 2017; Reichardt et al. 2018). Many *SBT1* genes have been implicated in biotic interactions, including symbiotic interactions (e.g. arbuscular mycorrhiza and nodulation) as well as pathogenic interactions with viruses, microbes, insects, and parasitic plants. It was proposed that the *SBT1* clade expanded by whole-genome and tandem duplications followed by neo-functionalization in response to the selection pressure from interaction partners (Taylor and Qiu 2017). This would explain the smaller size of the *SBT1* clade in Arabidopsis, a non-mycorrhizal and non-nodulating species. Neo-functionalization implies the specialization of SBT paralogs for substrate proteins specifically involved in the different biotic interactions. However, these substrate proteins have yet to be identified.

Direct evidence for the diversification of clade 1 *SBTs* in response to pathogen pressure was obtained for the cluster of 10 monophyletic *P69* genes on tomato chromosome 8, with individual paralogs contributing to plant defense against different pathogens (Homma et al. 2023; Zhang et al. 2024b). Host immune responses rely on P69B which activates the immune protease Rcr3 (Paulus et al. 2020). Many pathogens produce effector proteins inhibiting P69B to suppress immunity (Homma et al. 2023). Responding to the resulting selection pressure, paralogs evolved within tomato and across related Solanum species that show variation mainly at residues located at the protease/inhibitor interface, thereby escaping effector-mediated P69 inhibition (Homma et al. 2023).

Neo-functionalization with respect to substrate specificity is apparent in the *SBT3* clade, which is much larger in Arabidopsis, with 18 members compared to only 2 in tomato (Reichardt et al. 2018). Many of the Arabidopsis *SBT3* copies are found as clusters of tandemly arrayed, monophyletic paralogs indicating that they originate from tandem duplications in the Brassicales or Brassicaceae (Taylor and Qiu 2017). Substrates of clade 3 SBTs include PROSCOOPs, a large family of phytocytokine precursors. PROSCOOP12 and PROSCOOP20 are processed by different SBT3 members to release the corresponding bioactive SCOOP peptides (Yang et al. 2023). Interestingly, the PROSCOOP family is also restricted to Brassicaceae (Gully et al. 2019), suggesting that co-evolution with PROSCOOPs may have contributed to the expansion of the SBT3 clade.

#### Specialization with respect to mechanisms of regulation

Proteases must be tightly controlled, due to their irreversible impact on the structure and function of substrate proteins. This is achieved by diversification with respect to the developmental stage and cell type in which they occur, the subcellular compartments and conditions under which they are active, and the mechanisms by which proteolytic activity is terminated. SBTs show highly tissue-specific and/or stress-responsive expression patterns, likely resulting from sub- or neo-functionalization at the level of gene regulatory elements. They are produced as pre-pro-enzymes with an N-terminal signal peptide targeting the proteins to the secretory pathway. The prodomain serves dual functions, acting as a folding assistant and as an auto-inhibitor of enzymatic activity (Meyer et al. 2016). Cleavage of the prodomain is critical for enzyme activation and is an autocatalytic process. In tomatoes, SBT3 is controlled by pH (Meyer et al. 2016) and occurs late in the secretory pathway when the pH drops in the trans-Golgi, thereby preventing precocious enzyme activation. Since SBT activity is required also in earlier compartments of the secretory pathway, as well as in the apoplast (Stührwohldt et al. 2020b), other regulatory mechanisms for prodomain processing are likely to exist. Some SBTs are kept inactive, even after prodomain removal, by a self-inhibitory N-terminal peptide or a flexible β-hairpin occluding the active site. These SBTs require further processing and homodimerization, respectively, for activation (Janzik et al. 2000; Ottmann et al. 2009). SBTs also diversified with respect to the pH optimum for catalysis, ranging from pH 4 to 11. How all these factors relate to physiological function is still largely unresolved.

Protease activity can be terminated by inhibition, degradation, or sequestration. In addition to pathogen-derived inhibitory effector proteins (Homma et al. 2023), there are also endogenous SBT inhibitors that are related in structure and function to the SBT prodomain (Hohl et al. 2017). In Arabidopsis, this includes subtilisin propeptide-like inhibitor 1 (SPI-1), a potent inhibitor with inhibition and dissociation constants in the picomolar range (Hohl et al. 2017). However, which SBTs are targeted in vivo, and the physiological consequences of SPI-mediated inhibition, remain to be identified. The activity of AtS1P in clade SBT7 is regulated by the Serpin1 inhibitor (Ghorbani et al. 2016). Whether plant-specific SBTs in clades SBT1-5 are inhibited by other members of the large serpin family is still unknown. As other means of regulation, tomato subtilase P69B is degraded by two matrix metalloproteinases (Zimmermann et al. 2016), and active phytaspase (AtSBT3.8) is specifically removed from the apoplast by clathrin-mediated endocytosis (Trusova et al. 2019).

#### Specialization for specific processing sites

An emerging function of plant SBTs is their predominant role in the formation of peptide hormones and growth factors, particularly of the post-translationally modified signaling peptides as extracellular signals for cell-to-cell communication (Stührwohldt and Schaller 2019; Stintzi and Schaller 2022). The vast numbers of signaling peptides and SBTs in the plant apoplast suggest that mechanisms are in place both to prevent unwanted degradation of signaling peptides and to ensure the specificity of peptide precursor processing. Such mechanisms may have arisen by co-evolution of the signaling peptide and SBT families.

To ensure the specificity of precursor processing, SBTs evolved different modes of cleavage site recognition. Precise processing of the IDA precursor *(*a peptide controlling floral organ abscission*)* depends on multiple residues on either side of the cleaved bond. These residues are accommodated in the active site cleft of SBT4.13 with low selectivity, and it is the sum of many low-affinity interactions that ensures precise recognition of the cleavage site (Schardon et al. 2016). In contrast, the precursors of systemin and PSK (peptides controlling herbivore defense and flower drop in tomato, respectively) display aspartate residues at their processing sites. Phytaspases recognize these single aspartates in a highly specific manner, showing little selectivity for other residues around the cleavage site (Beloshistov et al. 2018; Reichardt et al. 2018; Reichardt et al. 2020). The TWS1 precursor (TWS1 is a peptide controlling embryonic cuticle development*)* is processed by SBT1.8 and SBT2.4, which act redundantly at the C-terminal cleavage site (Doll et al. 2020; Royek et al. 2022). Interestingly, SBT1.8 also cleaves at the N-terminus, but only when a neighboring tyrosine is sulfated. In this case, post-translational modification of this tyrosine marks the cleavage site for recognition by SBT1.8 (Royek et al. 2022). The opposite was observed for the precursor of CLE40 (a peptide controlling stem cell maintenance in the root apical meristem). proCLE40 is cleaved by three redundant SBTs at two sites, the first resulting in the release of the mature peptide, and the second producing an inactive CLE40 fragment (Stührwohldt et al. 2020a). Here, post-translational hydroxylation of a neighboring proline prevents cleavage at the second site, thereby contributing to the specificity of processing and CLE40 biogenesis (Stührwohldt et al. 2020a). In these examples, different modes of substrate recognition by the proteases, and post-translational modifications of the peptide precursors both contribute to the specificity of interaction.

Given the preceding discussion, we can only offer a partial explanation for why there are so many peptidases in plants. Hence, the question is still open, awaiting thorough investigations into the evolutionary forces that propel the expansion of SBT and other peptidase families.

## Dive Curated Terms

The following phenotypic, genotypic, and functional terms are of significance to the work described in this paper:

FBL17 Gramene: AT3G54650
FBL17 Araport: AT3G54650

UBC32 Gramene: AT3G17000
UBC32 Araport: AT3G17000

CRL Gramene: AT5G51020
CRL Araport: AT5G51020

ftsH3 Gramene: AT2G29080
ftsH3 Araport: AT2G29080

ABI1 Gramene: AT4G26080
ABI1 Araport: AT4G26080

CDC48 Gramene: AT3G09840
CDC48 Araport: AT3G09840

CLE40 Gramene: AT5G12990
CLE40 Araport: AT5G12990

RBR1 Gramene: AT3G12280
RBR1 Araport: AT3G12280

RGLG1 Gramene: AT3G01650
RGLG1 Araport: AT3G01650

SPL1 Gramene: AT2G47070
SPL1 Araport: AT2G47070

COP1 Gramene: AT2G32950
COP1 Araport: AT2G32950

DET1 Gramene: AT4G10180
DET1 Araport: AT4G10180

PIF3 Gramene: AT1G09530
PIF3 Araport: AT1G09530

PIF4 Gramene: AT2G43010
PIF4 Araport: AT2G43010

MAX2 Gramene: At2g42620
MAX2 Araport: At2g42620

SPL2 Gramene: Solyc12g049330
SPL2 Araport: Solyc12g049330

SNC1 Gramene: AT4G16890
SNC1 Araport: AT4G16890

CBL10 Gramene: AT4G33000
CBL10 Araport: AT4G33000

CRY1 Gramene: AT4G08920
CRY1 Araport: AT4G08920

FTSH11 Gramene: AT5G53170
FTSH11 Araport: AT5G53170

HOS15 Gramene: At5g67320
HOS15 Araport: At5g67320

UVR8 Gramene: AT5G63860
UVR8 Araport: AT5G63860

ABI5 Gramene: AT2G36270
ABI5 Araport: AT2G36270

BIG Gramene: AT3G02260
BIG Araport: AT3G02260

CRL1 Gramene: Os03g05510
CRL1 Araport: Os03g05510

DGS1 Gramene: Os03g0706900
DGS1 Araport: Os03g0706900

CUL1 Gramene: AT4G02570
CUL1 Araport: AT4G02570

PYL8 Gramene: AT5G53160
PYL8 Araport: AT5G53160

RAD51 Gramene: AT5G20850
RAD51 Araport: AT5G20850

HYPK Gramene: AT3G06610
HYPK Araport: AT3G06610

PTI Gramene: pattern-triggered immunity
PTI Araport: pattern-triggered immunity
